# Association of metformin monotherapy or combined therapy with cardiovascular risks in patients with type 2 diabetes mellitus

**DOI:** 10.1186/s12933-020-01202-5

**Published:** 2021-01-30

**Authors:** Tian Li, Rui Providencia, Nan Mu, Yue Yin, Mai Chen, Yishi Wang, Manling Liu, Lu Yu, Chunhu Gu, Heng Ma

**Affiliations:** 1 Department of Physiology and Pathophysiology, Fourth Military Medical University, No. 169 Changle West Rd, Xi’an, 710032 China; 2 Barts Heart Centre, St. Bartholomew’s Hospital, London, UK; 3 Department of Cardiovascular Medicine, Xijing Hospital, Fourth Military Medical University, Xi’an, 710032 China; 4 Department of Pathology, Xijing Hospital, Fourth Military Medical University, Xi’an, 710032 China; 5 Department of Cardiovascular Surgery, Xijing Hospital, Fourth Military Medical University, Xi’an, 710032 China

**Keywords:** Metformin, Type 2 diabetes mellitus, Mortality, Cardiovascular diseases, Myocardial ischemia, Microvascular events

## Abstract

**Background:**

Metformin is a first-line drug in type 2 diabetes mellitus (T2DM) treatment, yet whether metformin may increase all-cause or cardiovascular mortality of T2DM patients remains inconclusive.

**Methods:**

We searched PubMed and Embase for data extracted from inception to July 14, 2020, with a registration in PROSPERO (CRD42020177283). This study included randomized controlled trials (RCT) assessing the cardiovascular effects of metformin for T2DM. This study is followed by PRISMA and Cochrane guideline. Risk ratio (RR) with 95% CI was pooled across trials by a random-effects model. Primary outcomes include all-cause mortality and cardiovascular mortality.

**Results:**

We identified 29 studies that randomly assigned patients with 371 all-cause and 227 cardiovascular death events. Compared with untreated T2DM patients, metformin-treated patients was not associated with lower risk of all-cause mortality (RR: 0.98; 95%CI: 0.69–1.38; *P* = 0.90), cardiovascular mortality (RR: 1.13; 95% CI: 0.60, 2.15; *P* = 0.70), macrovascular events (RR: 0.87; 95%CI: 0.70–1.07; *P* = 0.19), heart failure (RR: 1.02; 95% CI:0.61–1.71; *P* = 0.95), and microvascular events (RR: 0.78; 95% CI:0.54–1.13; *P* = 0.19). Combination of metformin with another hypoglycemic drug was associated with higher risk of all-cause mortality (RR: 1.49; 95% CI: 1.02, 2.16) and cardiovascular mortality (RR: 2.21; 95% CI: 1.22, 4.00) compared with hypoglycemic drug regimens with no metformin.

**Conclusion:**

The combination of metformin treatment may impose higher risk in all-cause and cardiovascular mortality. This finding, at least in part, shows no evidence for benefits of metformin in combination in terms of all-cause/cardiovascular mortality and cardiovascular events for T2DM. However, the conclusion shall be explained cautiously considering the limitations from UK Prospective Diabetes Study (UKPDS).

## Background

Diabetes mellitus is an enormous public health issue worldwide [1]. It is estimated that 26, 9.4, and 91.8 million adults have diagnosed diabetes, undiagnosed diabetes, and prediabetes in United States, respectively [2]. Type 2 diabetes mellitus (T2DM) accounts for more than 90% of the cases and leads to vascular complications and causes profound psychological and physical distress to both patients and family members [3]. Metformin [4], although discovered in 1950s, is still recommended as a first-line oral glucose-lowering medication by the American Diabetes Association (ADA) and European Association for the Study of Diabetes (EASD) [1, 5, 6]. These endorsements are based on metformin’s ability to improve glycemic profile and reduce cardiovascular mortality, without the risk of causing hypoglycemia and/or body weight gains, which are side effects commonly observed in other antidiabetic drugs [7].

**Fig. 1:**
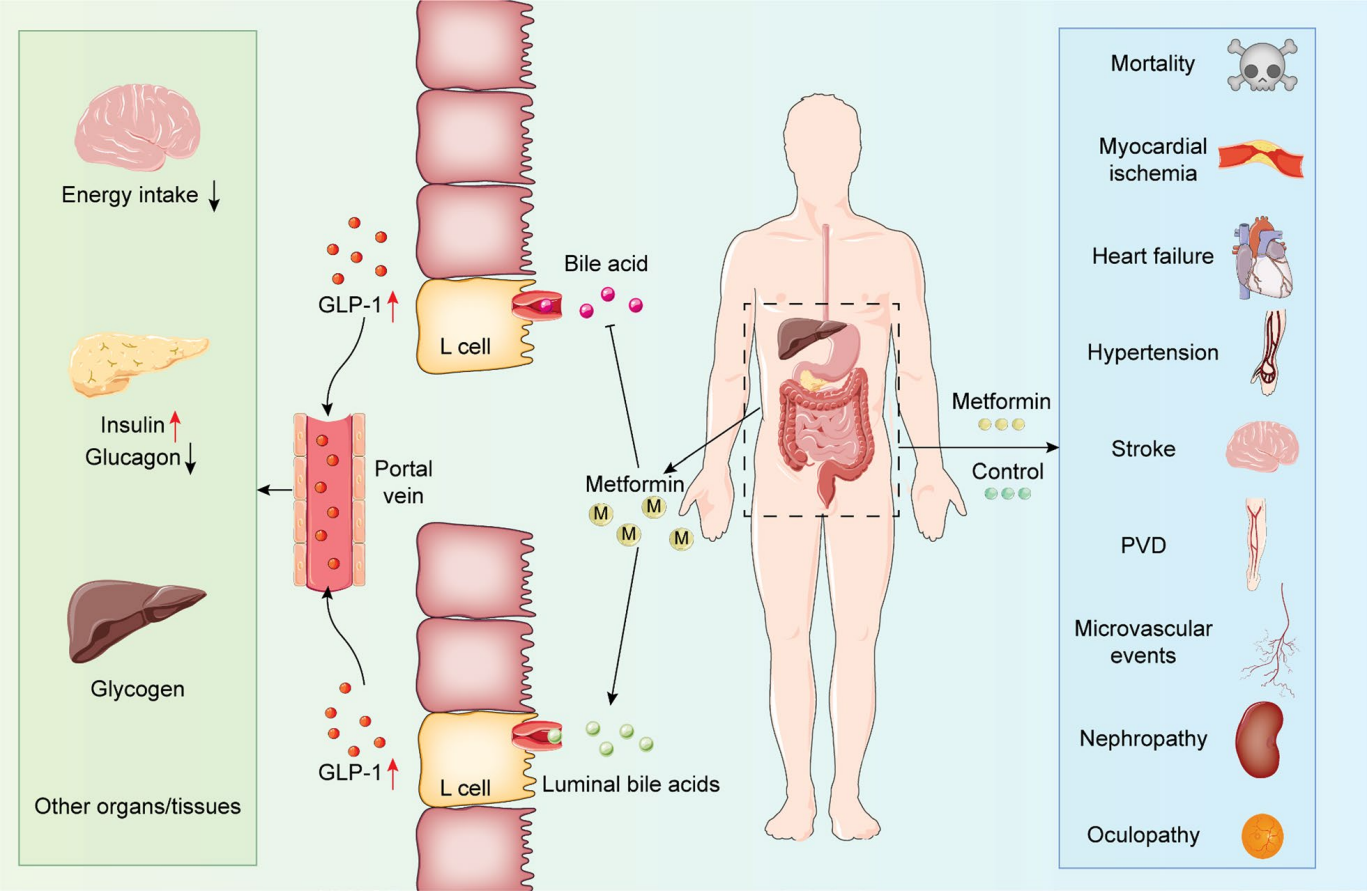
Snapshot of trial design and roles of metformin in glycometabolism. In the left side of illustration, metformin promotes the uptake of bile acid and luminal bile acid, and increases the levels of GLP-1, which further inhibits the energy intake of brain, pancreatic levels of glucagon, and hepatic glucagon degradation. Metformin also increases the secretion of insulin. In the right side of illustration, clinical usage of metformin has diverse roles on mortality, myocardial ischemia, heart failure, hypertension, stroke, *PVD* microvascular events, nephropathy, and ocupathy

It has been shown that metformin can reduce blood glucose levels in T2DM which provides vascular protection [8] (Fig. 1). Its cardio-cerebrovascular benefits are also elucidated by our previous animal research [9, 10], and narrative reviews [6, 11]. However, uncertainty remains on whether metformin confers cardiovascular protection in T2DM patients. Recent publications have shown that metformin can reduce [12, 13] or fail to reduce [14–16] the risk of cardiovascular disease in T2DM patients. The objective of this article is to determine whether metformin monotherapy or combined therapy is associated with more cardiovascular risks in patients with type 2 diabetes mellitus.

## Methods

### Study protocol

This article was in accordance to the Preferred Reporting Items for Systematic Reviews and Meta-Analyses (PRISMA) guideline [17] and registered on PROSPERO (CRD42020177283).

### Study selection and quality assessment

Two reviewers (Li and Mu) independently searched the Cochrane, PROSPERO, Joanna Briggs Institute (JBI), and INPLASY database, to avoid any duplicates in meta-analysis topics. We then searched PubMed and Embase from inception to July 14, 2020 by using medical subject headings (MeSH), Emtree, and text word with no language limitations (Additional file 1: Table S3). References of relevant reviews, editorials, and letters were also searched manually. We imported the publications into EndNote X9.1, removed duplicate records, excluded irrelevant literature, screened the titles/abstracts, and enrolled studies with detailed classification (Additional file 1: Table S4–S5). In addition, we also obtained full text and raw data from authors via email correspondence. Any inconsistency was forwarded to a third reviewer/cardiologist (Ma) for final decision. Eventually, the following information was extracted in a standardized Excel, including first author, journal, publication year, region, intervention/control group, sample size, age, male percentage, trial duration, diabetes duration, HbA1c (%), and data presentation.

### End points

All statistical tests (unless otherwise noted) were performed using two-tailed *t*-test and *P* < 0.05 was deemed statistically significant. Data were analyzed using STATA 16.0 (Stata Corp, College Station, TX, USA) and RevMan 5.3 (Nordic Cochrane Center, Copenhagen, Denmark). Dichotomous data were calculated as relative risks (RR) with 95% confidence intervals (CI). Effect size was performed by a forest plot including the overall effect size and its 95% CI. The weight of enrolled studies depended on the value of events of treatment group, events of control group, and total sample size. No statistical difference was defined where the 95% CI and null line intersected. Primary outcomes were defined as all-cause and cardiovascular mortality. Secondary outcomes included macrovascular events (myocardial ischemia, stroke, hypertension, and peripheral vascular diseases), heart failure, and microvascular events (Fig. 1).

### Risk of bias and heterogeneity analysis

The risk of bias calculation was applied according to the Cochrane guidelines [18]. The included trials were graded as low quality, high quality, or moderate quality based on the Cochrane criteria.

Heterogeneity was defined as variation between the study effect sizes that cannot be explained by sampling variability alone, including clinical, methodological, and statistical heterogeneity. Heterogeneity was assessed using chi-squared test and I^2^ test. Data were considered with high heterogeneous if chi-squared test yielded P < 0.10 and I^2^ > 50% [19]. Labbe and Galbraith plot were used for intuitive judgment of heterogeneity. For remaining circumstances, a random effect model was used for pooling the effect size to calculate for statistical heterogeneity.

## Results

### Literature search

Upon literature search, curation, and analysis, we did not find any duplicates in meta-analysis topics in the databases used. High agreement value of initial decisions on the inclusion of studies was indicated (κ = 0.817, 95% CI: 0.531–0.925). A total of n = 2095 articles were identified during the initial search, after excluding duplicate records (n = 338). 104 articles were retained after excluding 1991 records after title/abstract curation. Thereafter, we read full text and enrolled 34 articles for qualitative synthesis and identified 18 articles (that contained 29 clinical studies in total) for final quantitative synthesis (Additional file 1: Fig S1).

### Study characteristics

The characteristics of included studies for quantitative (18 articles, 29 studies) [20–37] and qualitative analysis (20 articles, 30 studies, 7372 patients) [21, 22, 26, 27, 38–53] are exhibited in Additional file 1: Table S1 and Table S2, respectively. Multiple subgroups within a single study were included for analysis.

Studies originated from North America, in Europe, in Asia, and in multiple countries spanning between 1991 and 2018. In relationship to our defined outcomes, 14 studies reported the outcome of all-cause mortality [22, 25–27, 29, 31, 33, 37], 6 studies reported cardiovascular mortality [26, 27, 31, 33, 37], 13 studies reported myocardial ischemia [20, 22–24, 27–29, 31–33, 36, 37], 4 studies reported stroke [22, 31, 37], 5 studies reported hypertension [21, 27, 30, 35], 5 studies reported peripheral vascular diseases [22, 29, 31, 37], 4 studies reported heart failure [22, 31, 37], 3 studies reported microvascular events [31, 37], 6 studies reported nephropathy [21, 28, 31, 34], and 2 studies reported oculopathy [31, 33].

### Assessment of risk of bias

Risk-of-bias assessments of selected articles are summarized in Additional file 1: Fig S2. Four articles had a low risk of bias while 14 articles had a moderate risk. Most studies were categorized as moderate risk due to the lack of random sequence generation and allocation concealment.

### All-cause mortality

**Fig. 2:**
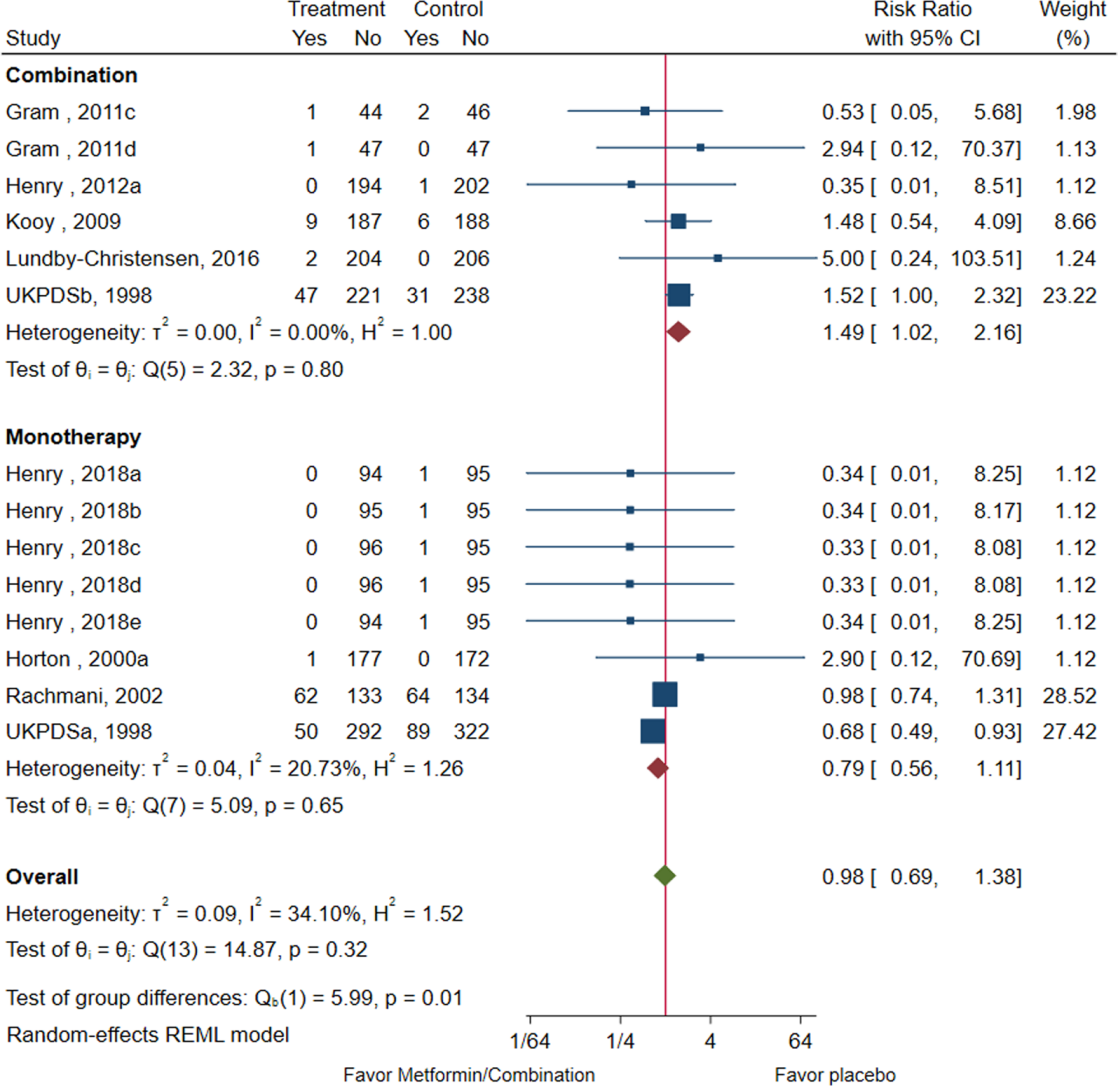
All-cause mortality among patients with metformin vs. placebo; and combination vs. placebo. Metformin treatment has no meaningful impact on all-cause mortality versus control, with a moderate heterogeneity. When used in conjunction with another antidiabetic drug, it significantly increased the all-cause mortality risk, compared to the antidiabetic drug alone

**Fig. 3:**
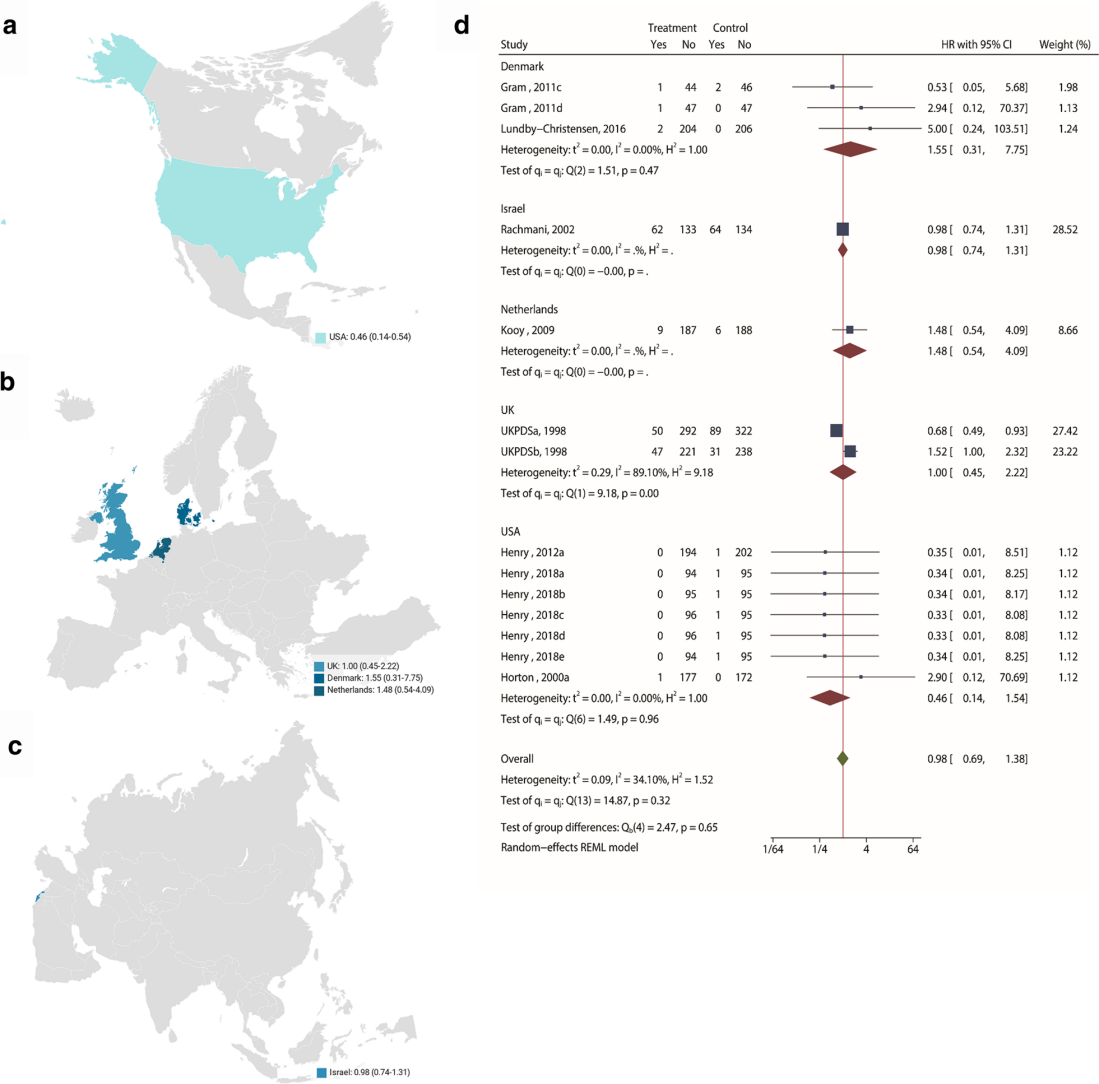
Choropleth map of all-cause mortality. The map reveals that regional difference of all-cause mortality in Denmark, Israel, Netherlands, UK, and USA. Metformin treatment has no meaningful impact on all-cause mortality versus control, in each country

Pooled analysis of 14 studies (n = 4375) [22, 25–27, 29, 31, 33, 37] demonstrates that metformin treatment has no meaningful impact on all-cause mortality (RR: 0.98; 95% CI: 0.0.69, 1.38; *P* = 0.90, Fig. 2) versus control, with a moderate heterogeneity (I^2^ = 34.1%, Fig. 2). The choropleth map reveals that regional difference of all-cause mortality in Denmark, Israel, Netherlands, UK, and USA. Metformin treatment has no meaningful impact on all-cause mortality versus control, in each country (Fig. 3). When used in conjunction with another antidiabetic drug, it significantly increased the all-cause mortality risk (RR: 1.49; 95% CI: 0.1.02, 2.16), compared to the antidiabetic drug alone (Fig. 2). Subgroup analysis showed that the combinatorial increase in all-cause mortality risk was not due to diabetes duration, trial duration, nor metformin dose (Fig. 2, Additional files 1: Fig S5–8).

The heterogeneity is significant in groups of newly diagnosed and ≥ 12 months. Their overall effects are all insignificant (*P* > 0.05) except the monotherapy/combination subgroup, showing combination therapy may increase the all-cause mortality (RR: 1.49; 95% CI: 1.02, 2.16, Fig. 2) with low heterogeneity (I^2^ = 0, Fig. 2). The cumulative meta-analysis suggests that the 95% CI is narrower with the increase of year and study size generally (Additional files 1: Fig S9 and Fig S10).

The Labbe plot show no significant heterogeneity (Additional file 1: Fig S11). The meta-regression by Bubble plot also reveals no significant heterogeneity of the year (I^2^ = 38.24%, R2 = 0, *P* = 0.552, Additional file 1: Fig S12) and study size (I^2^ = 27.34%, R2 = 0, *P* = 0.859, Additional file 1: Fig S13). Additional file 1: Fig S14 is contour-enhanced funnel plot that shows no significant publication bias.

### Cardiovascular mortality

**Fig. 4:**
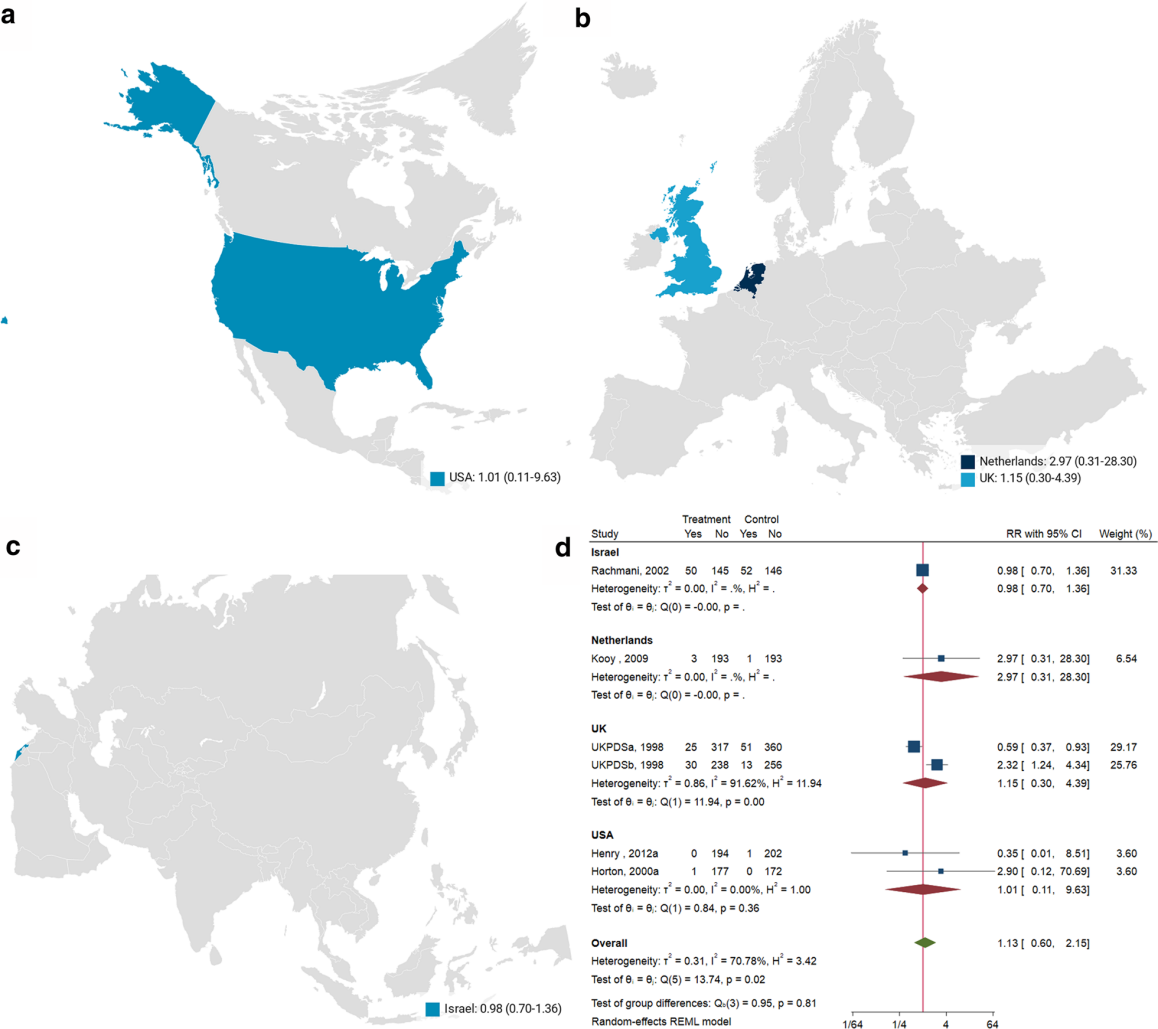
Choropleth map of cardiovascular mortality. The map reveals that regional difference of cardiovascular mortality in Israel, Netherlands, UK, and USA. Metformin treatment has no meaningful impact on cardiovascular mortality versus control, in each country

**Fig. 5:**
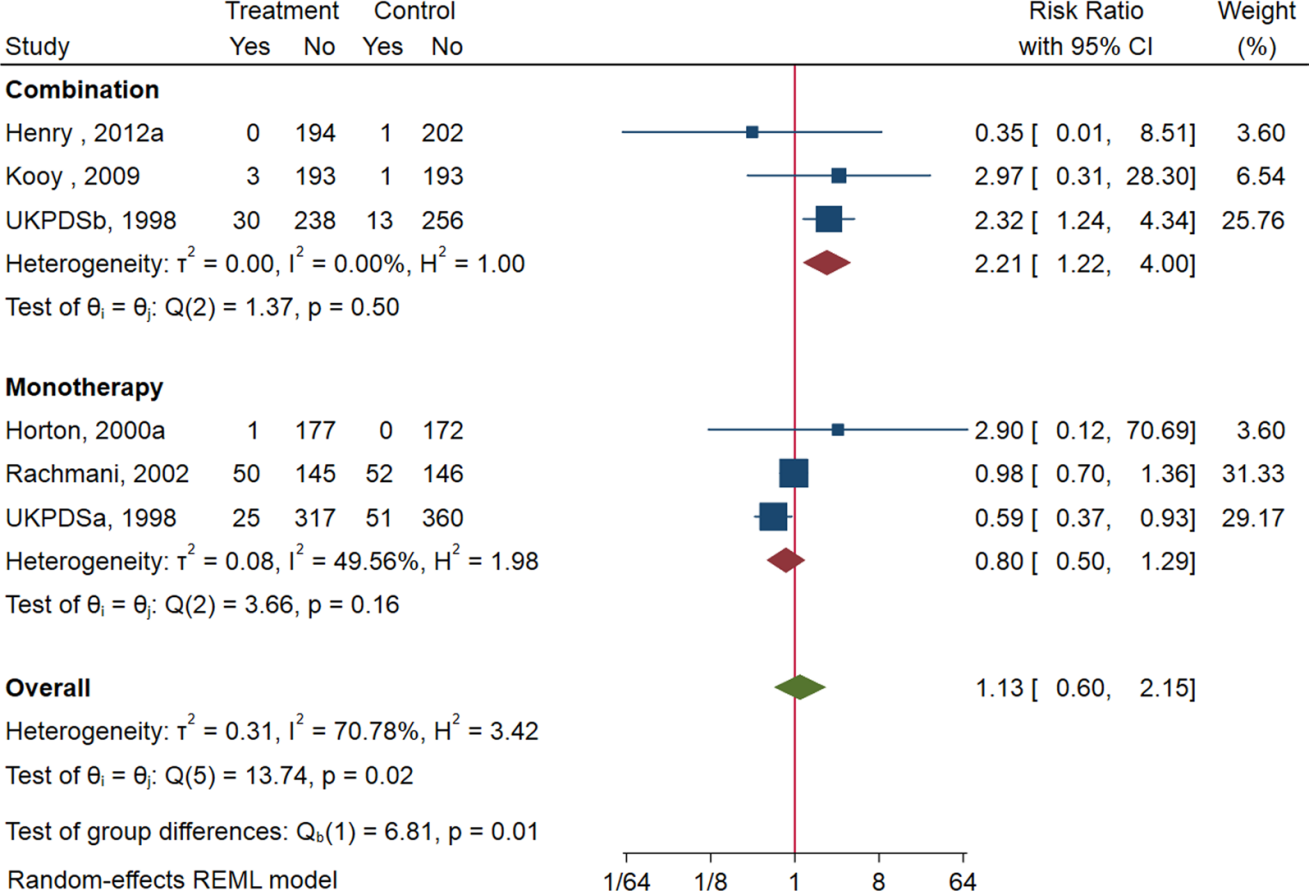
Cardiovascular mortality among patients with metformin vs. placebo; and combination vs. placebo. Metformin treatment has no meaningful actions on cardiovascular mortality versus control, with a high heterogeneity. However, metformin in combination increases the cardiovascular mortality versus metformin alone

Pooled analysis of 6 studies (n = 2820) [26, 27, 31, 33, 37] reveals that metformin treatment has no meaningful actions on cardiovascular mortality (RR: 1.13; 95% CI: 0.60, 2.15; P = 0.70, Additional file 1: Fig S4) versus control, with a high heterogeneity (I^2^ = 68.2%, Additional file 1: Fig S4). Choropleth map reveals that regional difference of cardiovascular mortality in Israel, Netherlands, UK, and USA. Metformin treatment has no meaningful impact on cardiovascular mortality versus control, in each country (Fig. 4). Subgroup analysis of diabetes duration, trial duration, metformin dose, and monotherapy/combination are shown (Additional file 1: Fig S15). The heterogeneity is significant in groups of monotherapy, newly diagnosed, ≥ 12 months. Their overall effects are all insignificant (*P* > 0.05) except the monotherapy/combination subgroup, showing combination therapy may increase cardiovascular mortality (RR: 2.21; 95% CI: 1.22, 4.00, Fig. 4) of T2DM patients with low heterogeneity (I^2^ = 0, Fig. 5, Additional file 1: Fig S15).

### Macrovascular events

Pooled analysis of 13 studies (n = 4150) [20, 22–24, 27–29, 31–33, 36, 37] shows that metformin treatment may not reduce the risk of myocardial ischemia (RR: 0.87; 95% CI: 0.70, 1.07; P = 0.19, Additional file 1: Fig S16) versus control, with a low heterogeneity (I^2^ = 7.14%, Additional file 1: Fig S16). Pooled analysis of 4 studies (n = 2051) [22, 31, 37] reveals that metformin treatment may not reduce the risk of heart failure (RR: 1.02; 95% CI: 0.0.610, 1.71; *P* = 0.95) versus control, with a low heterogeneity (I^2^ = 0, Additional file 1: Fig S17). There is also no statistical significance for the effect size of stroke (RR: 0.89; 95% CI: 0.53, 1.47; *P* = 0.64; I^2^ = 7.52%, Additional file 1: Fig S18) [22, 31, 37], hypertension (RR: 1.54; 95% CI: 0.52, 4.61; *P* = 0.44; I^2^ = 35.94%, Additional file 1: Fig S19) [21, 27, 30, 35], and peripheral vascular diseases (RR: 0.98; 95% CI: 0.60, 1.60; P = 0.93; I^2^ = 0, Additional file 1: Fig S20) [22, 29, 31, 37].

### Microvascular events

Pooled analysis of 3 studies (n = 1680) [31, 37] reveals that metformin treatment may not reduce the risk of microvascular events (RR: 0.78; 95% CI: 0.54, 1.13; *P* = 0.19, Additional file 1: Fig S21) versus control, with a low heterogeneity (I^2^ = 0, Additional file 1: Fig S32). There are also no There is also no effect on nephropathy (RR: 1.03; 95% CI: 0.61, 1.74; *P* = 0.92; I^2^ = 0, Additional file 1: Fig S22) and oculopathy (RR: 1.30; 95% CI: 0.47, 3.59; *P* = 0.61; I^2^ = 0, Additional file 1: Fig S23).

## Discussion

### Main findings

Our results based on 29 studies demonstrate that compared with other antidiabetic medications, metformin may not reduce mortality, macrovascular and microvascular events of T2DM people. Metformin in combination with sulfonylurea was associated with higher risk of all-cause and cardiovascular mortality. This finding, at least in part, shows no benefits of metformin in combination in reducing mortality and cardiovascular risk, and raises a word of caution regarding combination therapy with metformin and other antidiabetic agents.

### Interpretation

Among the curated cohort, we observed no statistical benefit of metformin compared to control group. The majority of data came from the UK Prospective Diabetes Study (UKPDS) [37], a trial concerning the effectiveness and safety of treatments for type 2 diabetes. The weight of all-cause and cardiovascular mortality from UKPDS is 50.64 and 54.93%, respectively. However, the conclusion shall be explained with caution due to their limitations. (UKPDSa) concluded metformin + diet reduces the all-cause and cardiovascular mortality, and myocardial ischemic risk [37], whereas UKPDSb, revealed that metformin + sulfonylurea increase all-cause and cardiovascular mortality vs sulfonylurea alone [37].

Besides, we also noticed Palmer’s conclusion [54] that metformin (alone or in combination) does not increase the risk of cardiovascular or all-cause mortality. His work is a network meta-analysis that included 301 clinical trials, which has a different methodology and inclusion/exclusion criteria. Palmer’s work was also partly questioned by Rodriguez-Gutierrez et al. [55]. Thus, future works are needed to elucidate this problem.

Included UKPDSb subgroup may explain why our conclusion is different from Selvin’s work [56]. Saenz’s work [57] also suggested metformin prevent some vascular complications, and mortality whereas it was withdrawn in 2015 as a result of multiple changes like new publications, methods and other standards. Pooling conclusion is partly in accordance with Griffin’s work [14], but our results included updated literature and draw a conclusion of the increased risk of metformin combination in all-cause and cardiovascular mortality.

The harmful effects of the combination of metformin remain elusive. For increased all-cause mortality (RR: 1.49, 95% CI: 1.02, 2.16), 4 studies compared metformin as an add-on therapy in patients receiving insulin [22, 29, 31], 1 in sulphonylurea [37], and 1 in dapagliflozin [26]. For all-cause mortality, the UKPDSb occupies the most weight, 23.22% (Additional file 1: Fig S3). For increased cardiovascular mortality (RR: 2.21, 95% CI: 1.22, 4.00), 3 studies compared metformin as an add-on therapy in patients receiving insulin [31], sulfonylurea [37], or dapagliflozin [26], respectively. For cardiovascular mortality, the UKPDSb also occupies a weight 25.76% (Additional file 1: Fig S4). Boussageon et al. [58] indicated the methodological weaknesses of UKPDS, thereby it shall be cautious to make metformin prohibited.

Han and colleagues included 40 studies comprising 1,066,408 patients and found that metformin reduces cardiovascular mortality, all-cause mortality and cardiovascular events in coronary artery diseases (CAD) patients [13]. For myocardial infarction patients and CAD patients without T2DM, metformin has no significant effect of reducing the incidence of cardiovascular events. These reasons may lead to the inconsistence between our and Han’s conclusion (beneficial effects of metformin): (1) The enrolled criteria are patients with CAD whereas T2DM (with or without CVD). The inconsistence of two kinds of population may contribute to difference. (2) Our study enrolled RCT (which is considered as clinical studies of highest quality of Cochrane Collaboration) only, whereas Han and colleagues enrolled both RCT and cohort studies, which may lead to more enrolled patients. (3) Our criteria for “Intervention (I)” includes metformin and its analogue whereas Han’s study enrolled metformin only. In short, our conclusion is based on the pooling analysis of all enrolled RCT and we would like to show this conclusion with all cardiologists, endocrinologists, statisticians, and evidence-based scientists for further discussion.

Recent studies also praise the use of metformin for cardioprotective purpose. Four hundred and twelve patients with T2DM were randomized to 18 months of metformin or placebo in addition to open‑labelled insulin. Hansen et al. found that eighteen months of metformin treatment in combination with insulin compared with insulin alone increased early drop in orthostatic blood pressure indicating an adverse effect of metformin on cardiovascular autonomic neuropathy independent of vitamin B12, methyl malonic acid [59]. One study consisted of 21,996 individuals (19,990 metformin users and 2006 sulfonylurea users). Metformin use was associated with lower risk for all-cause mortality (hazard ratio [HR], 0.48; 95% CI, 0.40–0.58), cardiovascular events (HR, 0.67; 95% CI, 0.52–0.86), and major hypoglycemic episodes (HR, 0.14; 95% CI, 0.09–0.20; *P* < 0.001) when compared with sulfonylureas [60], which is partly corresponding to our results (Figs. 2 and 5). Besides, Luo and colleagues reported that metformin was shown in pre-clinical and clinical studies, to lower the cardiovascular events in diabetes patients. Growing evidence suggests that metformin has a protective effect on coronary artery beyond its hypoglycemic effects. And metformin provides an alternate/additional therapeutic option for primary and secondary prevention of CAD in diabetes and non-diabetics alike [61]. Luo’s paper is a narrative review that enrolled both animal and clinical evidence, the research method is different from meta-analysis, which is considered as evidence of highest quality by Cochrane Collaboration. Authors may review and select literature subjectively, which meet their personal standard in a narrative review. These explanation above are possible reasons that cause the difference of conclusion vs our results in the paper. Besides, Campbell and colleagues reported that metformin users also had reduced cancer compared to non-diabetics (RR 0.94, 95% CI 0.92–0.97) and CVD compared to diabetics receiving non-metformin therapies (HR 0.76, 95% CI 0.66–0.87) or insulin (HR = 0.78, 95% CI 0.73–0.83). To be specific, they showed that diabetic people taking metformin had a lower rate of developing any cancer compared with the general population, and had a lower risk of developing colorectal, breast, and lung cancer compared with diabetics managing their diabetes through non-metformin therapies after adjusting for disease control [62]. Diabetics taking metformin have a lower rate of all-cause mortality than non-diabetic people and the general population [62].

Notably, the use of sodium-glucose co-transporter-2 (SGLT2) inhibitors was shown to reduce the risk of all-cause mortality, cardiovascular mortality, major kidney outcomes, and major adverse cardiovascular events, with and without concomitant metformin use [63]. Moreover, metformin has also been reported to have a beneficial effect on CVD independent of blood cholesterol [64, 65]. Luo et al. reported that atorvastatin/metformin combination therapy attenuates atherosclerotic plaques more effectively than monotherapy, however, metformin added to atorvastatin therapy has no additional lipid-lowering effect [64]. We also make an additional analysis for the association between metformin and lipid (Additional file 1: Fig S24) and corresponding systematic review.

Strikingly, the conclusion seems to be opposed to previous publications. Based on previous studies and hypothesis, the following explanations may address this question. (1) The study type is different. Previous publications are most animal research or cohort studies. Take melatonin for example, it is a protective drug of cardiovascular effects in vitro and in vivo whereas it has an unfavorable effect in clinical trials [66, 67]. (2) The pooling effect size is a summary of 95% CI and some enrolled studies in this meta-analysis show a survival/cardiovascular benefits, not for a single study. (3) Different eligibility criteria in those meta-analysis articles contribute to different enrolled population. Maybe this article included only trials while other reviews may have included non-trial data which probably had more positive results for metformin?

### Strength and limitations

Firstly, our meta-analysis was performed by a Cochrane Member and supervised by strict quality control evaluated by Cohen's kappa coefficient (κ = 0.817, 95% CI: 0.531–0.925). Secondly, we attempted to be as inclusive and transparent in this manuscript in terms of our methods including all origin of software and websites. Thirdly, we refined our analyses on Patient, Intervention, Control, Outcomes, and Study design in accordance to the PICOS principle. Specifically, we used 4–5 subgroups for Intervention analysis, sub-divided controls into placebo/diet and antidiabetics, and refined the outcomes into 11 types. Fourth, we eliminated data of ‘Double zero incident’ (the events number are 0 in both intervention and control group) per Cochrane Handbook which in previous studies the assumption skew the results. Raw data that were not included in original studies were obtained with authors’ consent via email correspondence.

Despite our best attempt, we acknowledge there are certain limitations in our study design. Firstly, although PubMed and Embase merely are researched in recent meta-analysis with high quality, we did not search other databases such as Cochrane Library, Web of Science, ClinicalTrials.gov, as PubMed and Embase included almost all databases and we formulate a retrieval strategy as per a tradeoff decision between recall ratio and precision ratio [68, 69]. Secondly, we were unable to connect with the authors of Sullivan’s study that contained a large sample size (Metformin + Placebo/Diet + Placebo) [51]. Thirdly, we did not include cohort studies, where most showed positive benefits for metformin, in fear of introducing selection bias [14, 58]. Fourthly, at least half of the study weight in the all-cause mortality outcome comes from the UKPDS study (50.63% of the weight), that has endless limitations such as selection bias of overweight patients, and enrolling patients 25 years ago. In practice, diabetic induced complications and mortality were significantly higher in 1990s than today.

Lastly, most excluded studies have unclear/poorly defined risks probably due to unreported random sequence generation and allocation concealment. And some RCT have a small sample size (< 50) that were conducted at single-centers.

### Implications

Despite misgiving [70], meta-analysis remains provides us insight to evidence-based medicine in patients currently approved by the Cochrane Collaboration. Metformin remains to be recommended as the first-line treatment for T2DM, and does confer protection against cancer and aging [71]. Although there is a large number of patients worldwide currently taking metformin, it is currently impossible to assess the side effects of metformin on cardiovascular events given that there are no large, double blind, placebo-controlled, metformin T2DM trial with cardiovascular outcome as endpoint [14].

Our results show that metformin increases the all-cause and cardiovascular mortality when combined with other antidiabetic agents (mainly sulfonylureas). Based on our findings, it should be recommended that metformin should be prescribed as a monotherapy and all combinatorial usage (with DPP-4, GLP-1) should be cautioned. Compared with other antidiabetics, metformin still should be recommended based on its low increased risk of the macrovasuclar, microvascular events, and heart failure [14, 58].

## Conclusions

Our results suggest that metformin may not reduce the mortality, macrovascular and microvascular events when used in conjunction with other antidiabetic prescriptions. This situation is somewhat reminiscent of rosiglitazone. In 2008, the FDA guidelines clearly stated that non-insulin hypoglycemic agents will not receive approval unless cardiovascular risk is assessed. Yet FDA voted to ease but not completely lift the restrictions on rosiglitazone in 2013 [72]. What about metformin? Although further large sample RCT are warranted, our meta-analyses cautiously recommend against, at least in part, the first-line use of metformin in combination therapy in T2DM due to potential survival and cardiovascular complications. However, the conclusion should be explained with caution considering the high weight of UKPDS that has many limitations.

## Supplementary Information

**Additional file 1.** Additional figures and tables.

## Data Availability

The analyzed datasets during the current study are available from the corresponding author on reasonable request.

## Abbreviations

T2DM: Type 2 diabetes mellitus
ADA: American Diabetes Association
EASD: European Association for the Study of Diabetes
PRISMA: Preferred Reporting Items for Systematic Reviews and Meta-Analyses
JBI: Joanna Briggs Institute
MeSH: Medical subject headings
RR: Relative risks
CI: Confidence intervals
UKPDS: UK Prospective Diabetes Study
