# Supplementary material for: Association of metformin monotherapy or combined therapy with cardiovascular risks in patients with type 2 diabetes mellitus
Source: Cardiovasc Diabetol. 2021 Jan 30;20:30. doi: 10.1186/s12933-020-01202-5 (PMC7847575; doi:10.1186/s12933-020-01202-5)
Supplement: Supplementary file 1 — Additional file 1. Additional figures and tables. [file 12933_2020_1202_MOESM1_ESM.docx]

**Additional files**

1. **Figures**

Fig S1-S24

1. **Tables**

Table S1. Characteristics of enrolled studies for quantitative analysis.

Table S2. Characteristics of enrolled studies for qualitative analysis.

Table S3. Search strategy.

Table S4. Inclusion or exclusion Criteria

Table S5. Reasons for excluded literature

Fig S1. PRISMA flow diagram of literature retrieval.


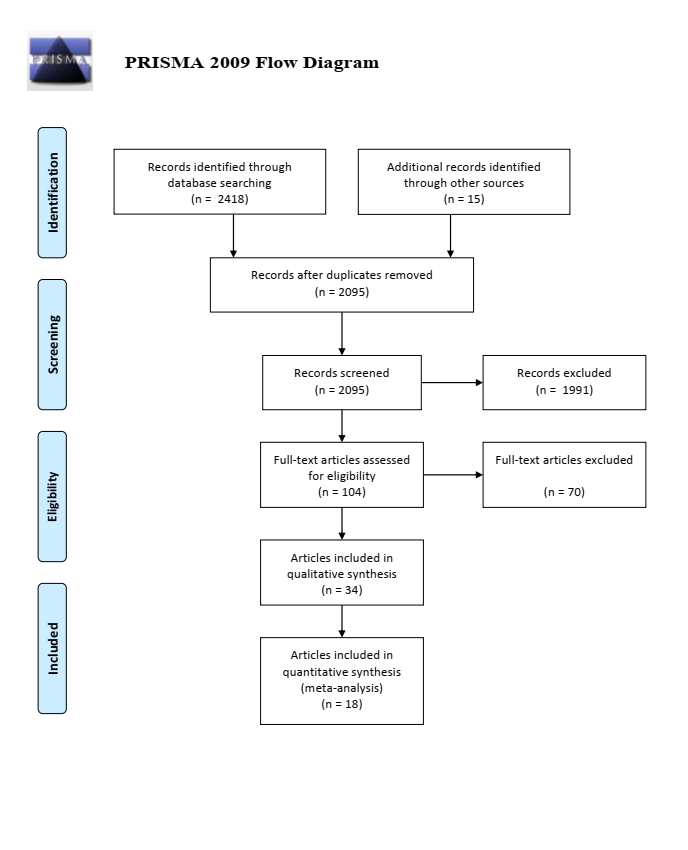


Fig S2. Risk of bias of included studies


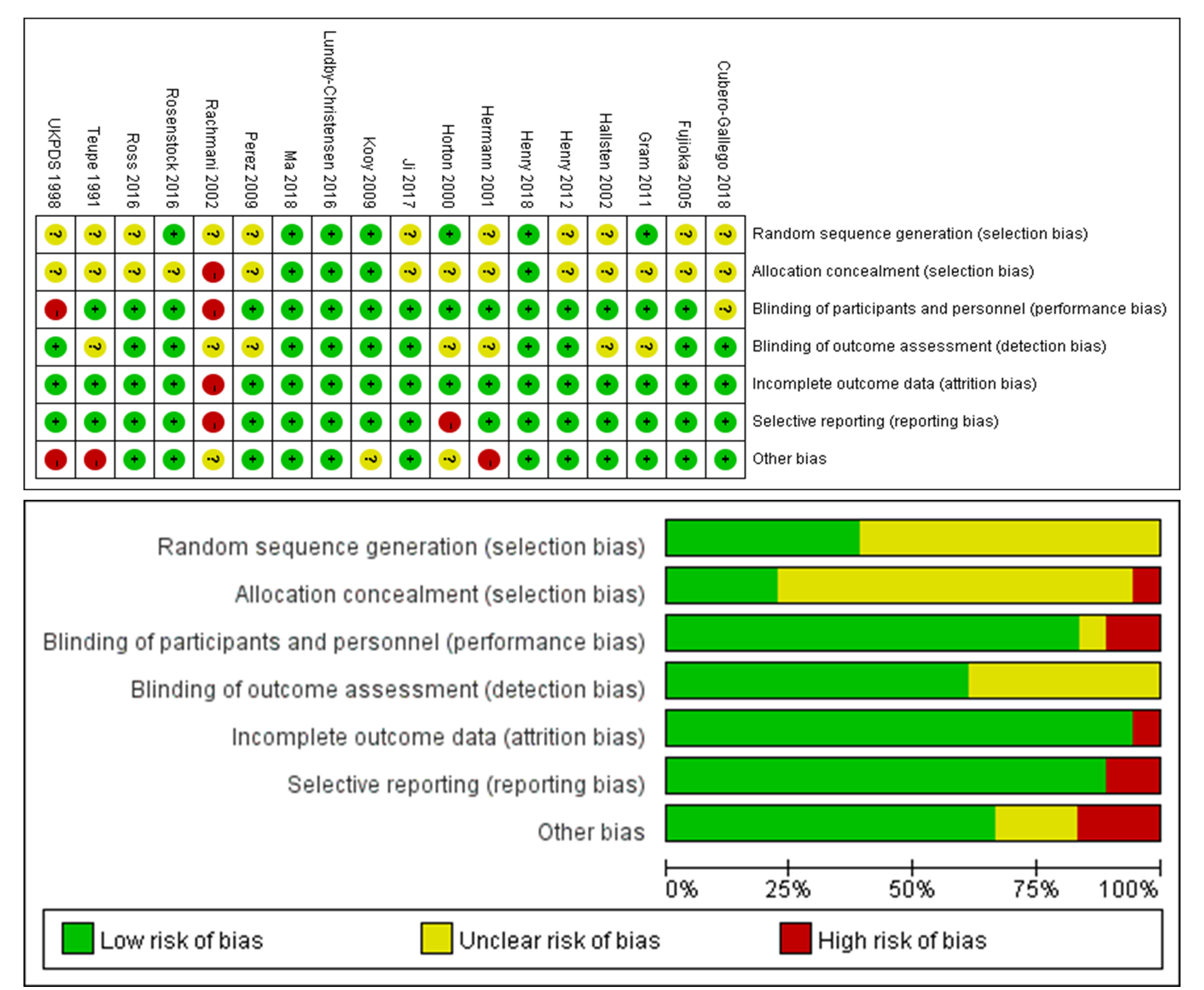


Fig S3. All-cause mortality among patients with metformin vs control.


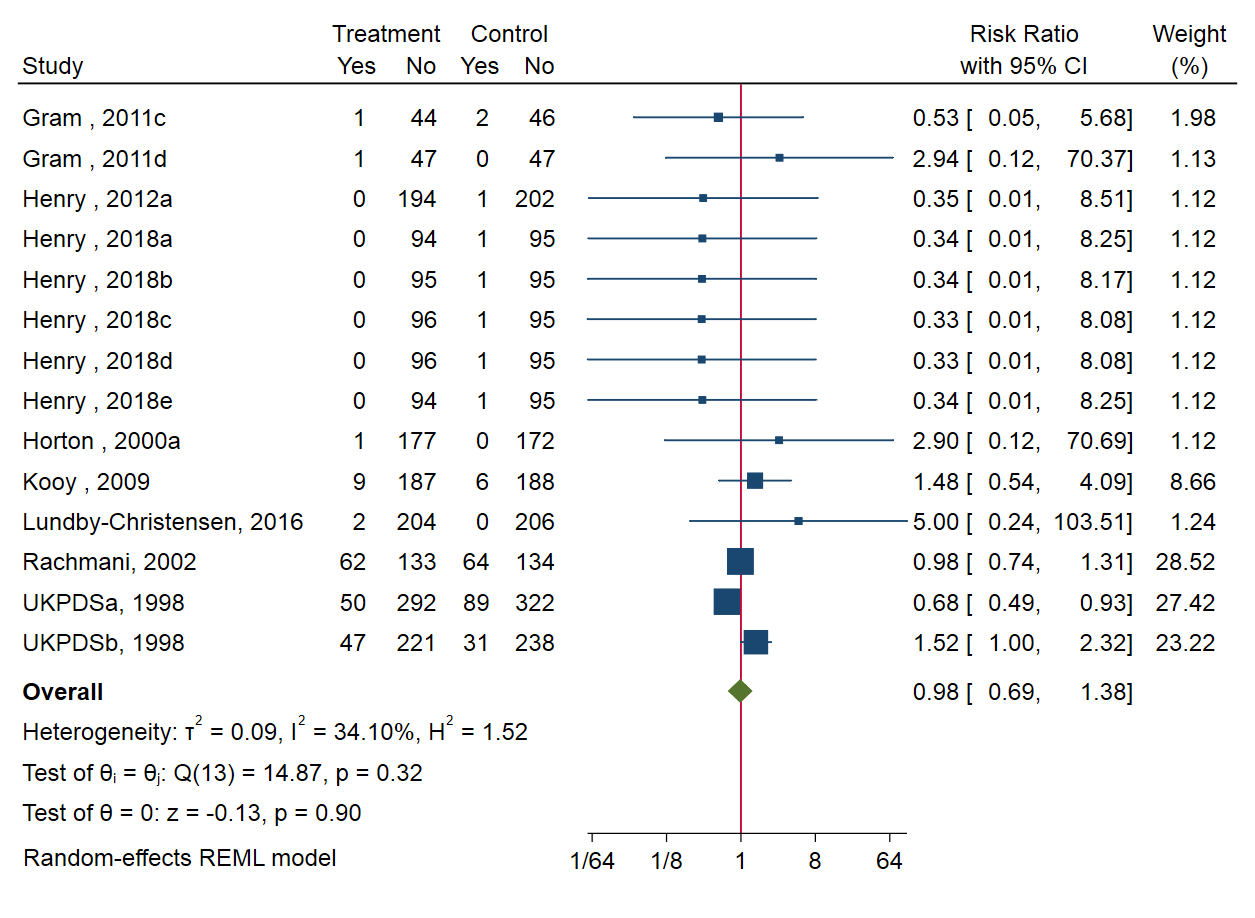


Fig S4. Cardiovascular mortality among patients with metformin vs control.


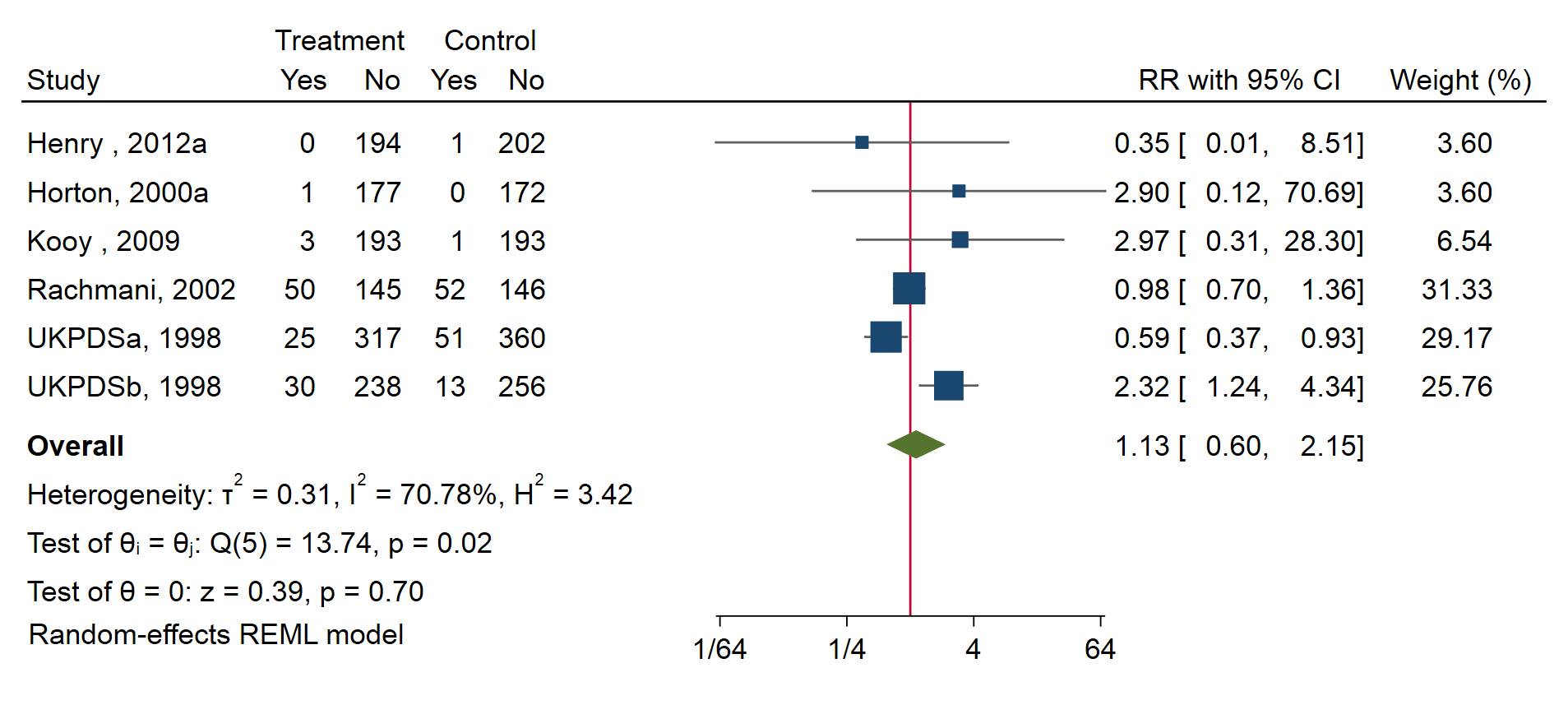


Fig S5. Subgroup analysis of all-cause mortality.


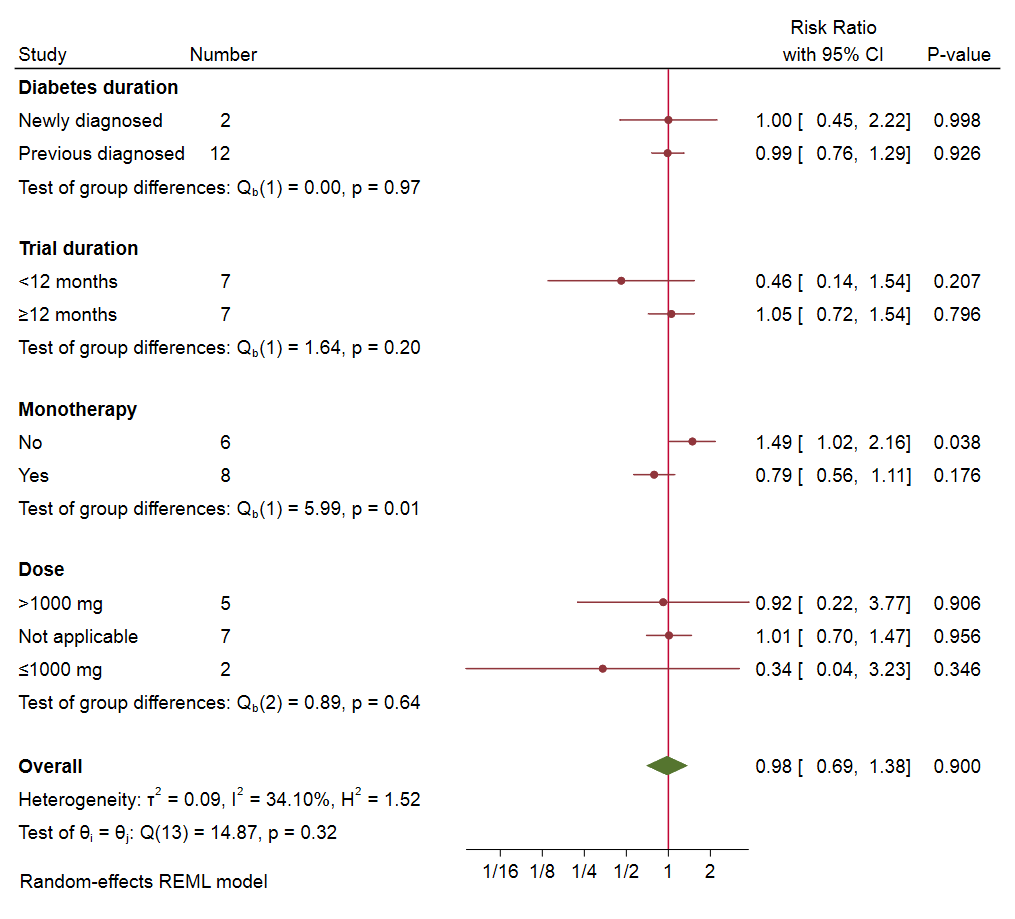


Fig S6. Diabetes duration subgroup analysis of all-cause mortality


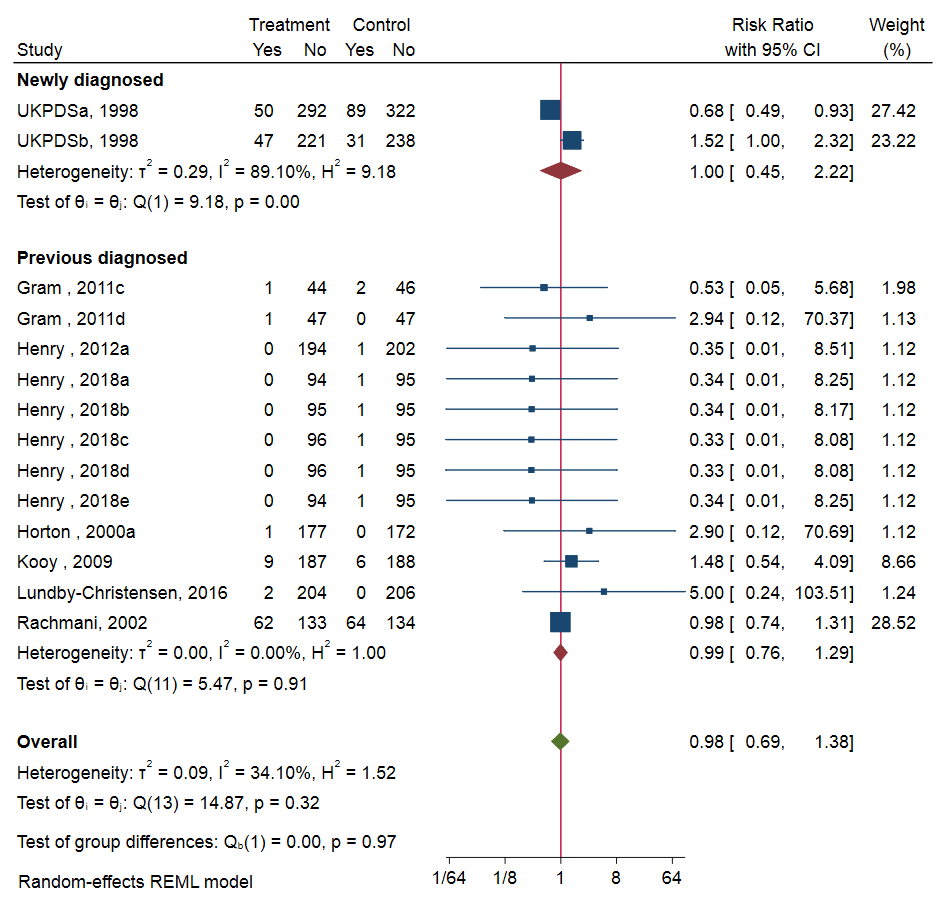


Fig S7. Metformin dose subgroup analysis of all-cause mortality


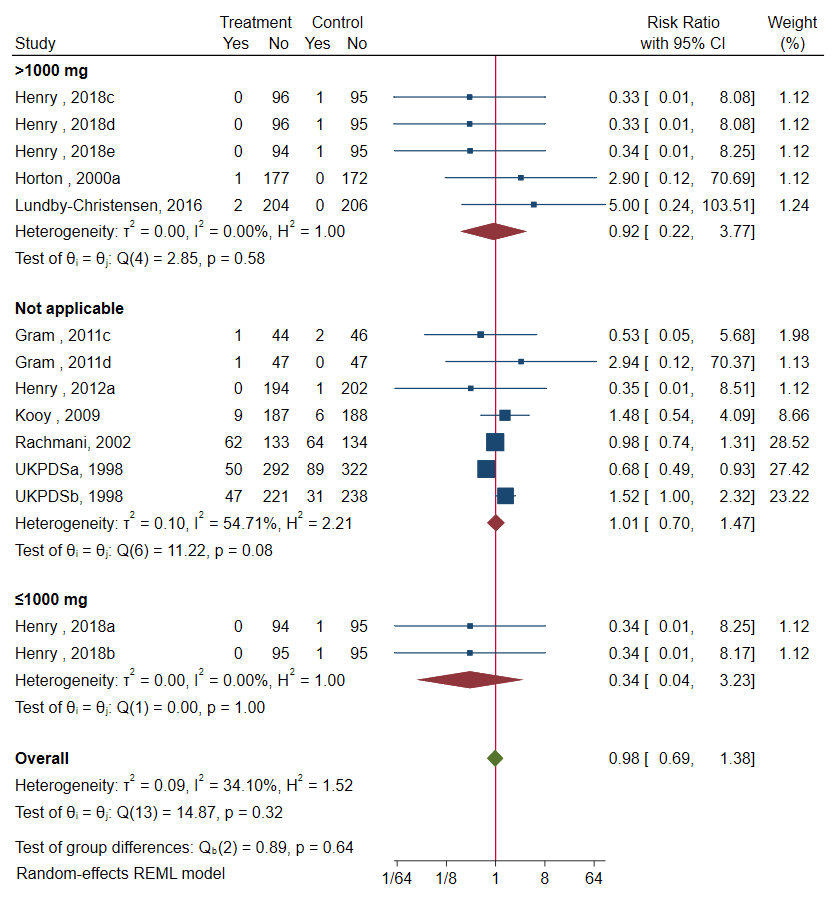


Fig S8. Trial duration subgroup analysis of all-cause mortality.


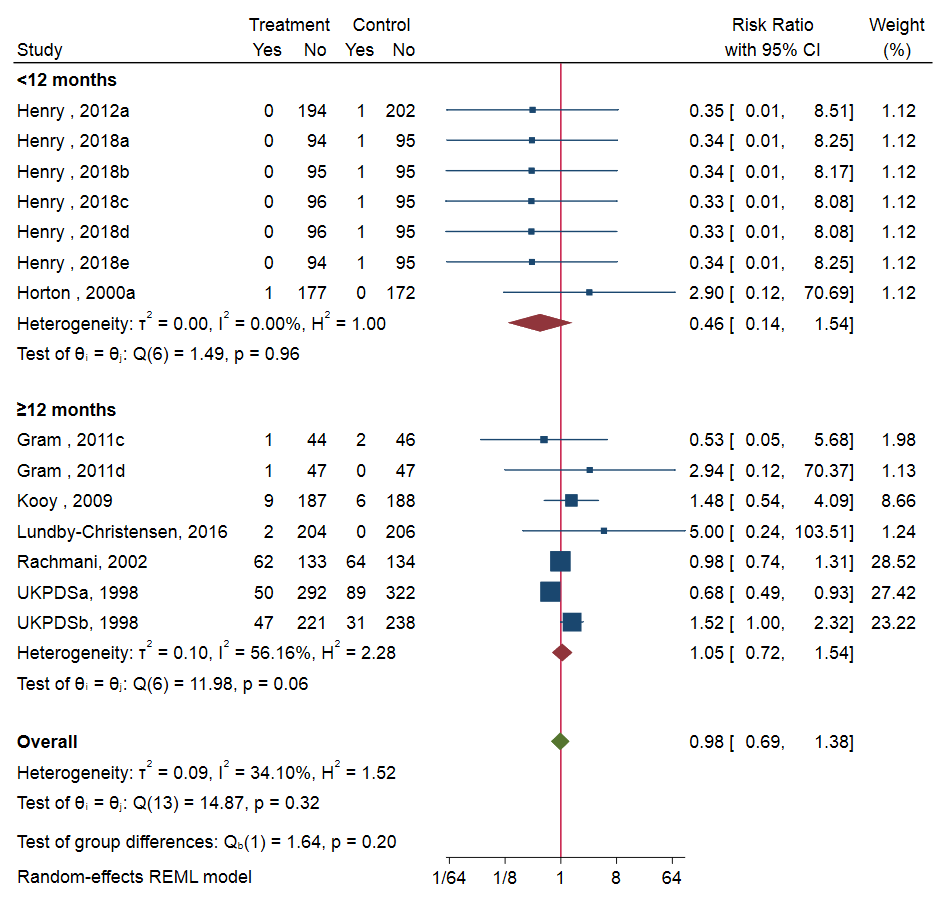


Fig S9. Cumulative meta-analysis of publication year of all-cause mortality.


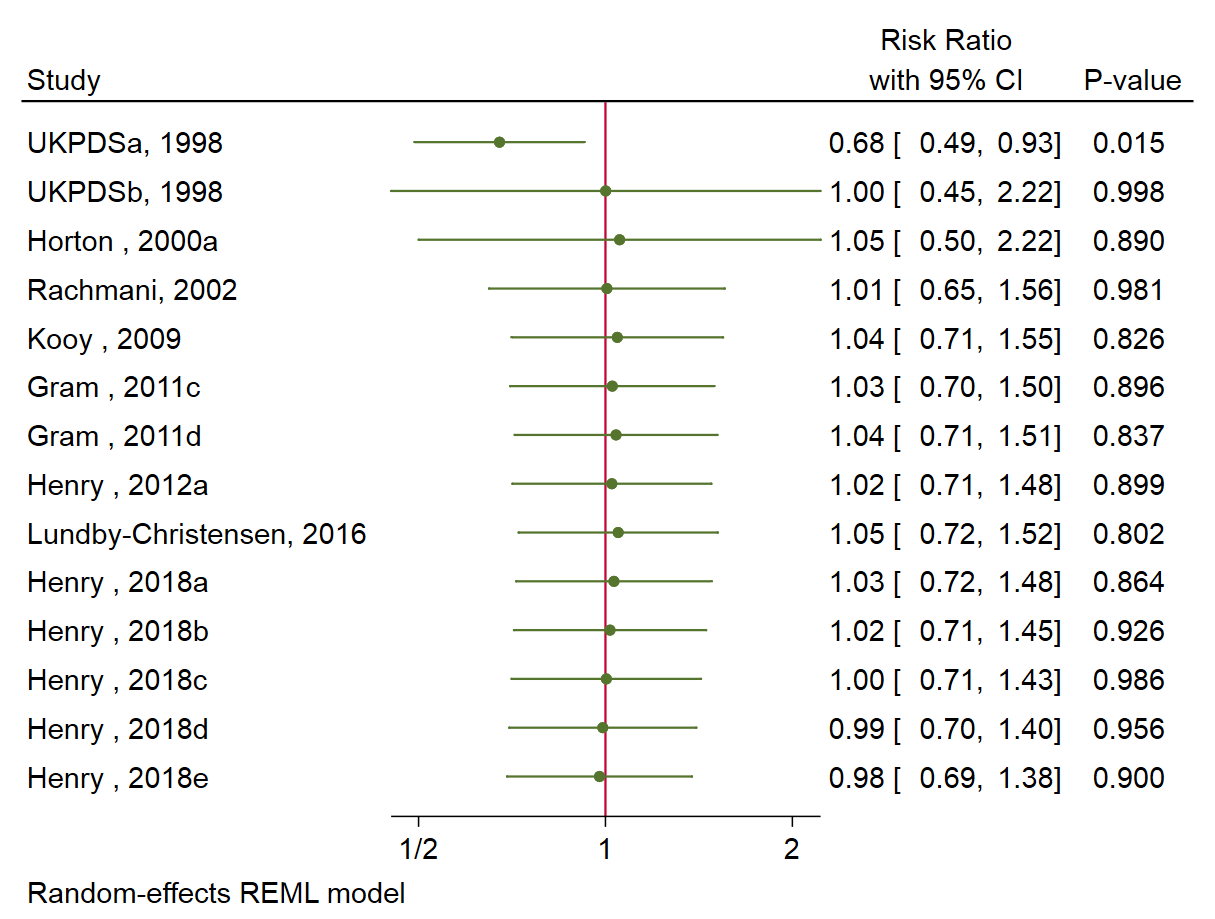


Fig S10. Cumulative meta-analysis of study size of all-cause mortality.


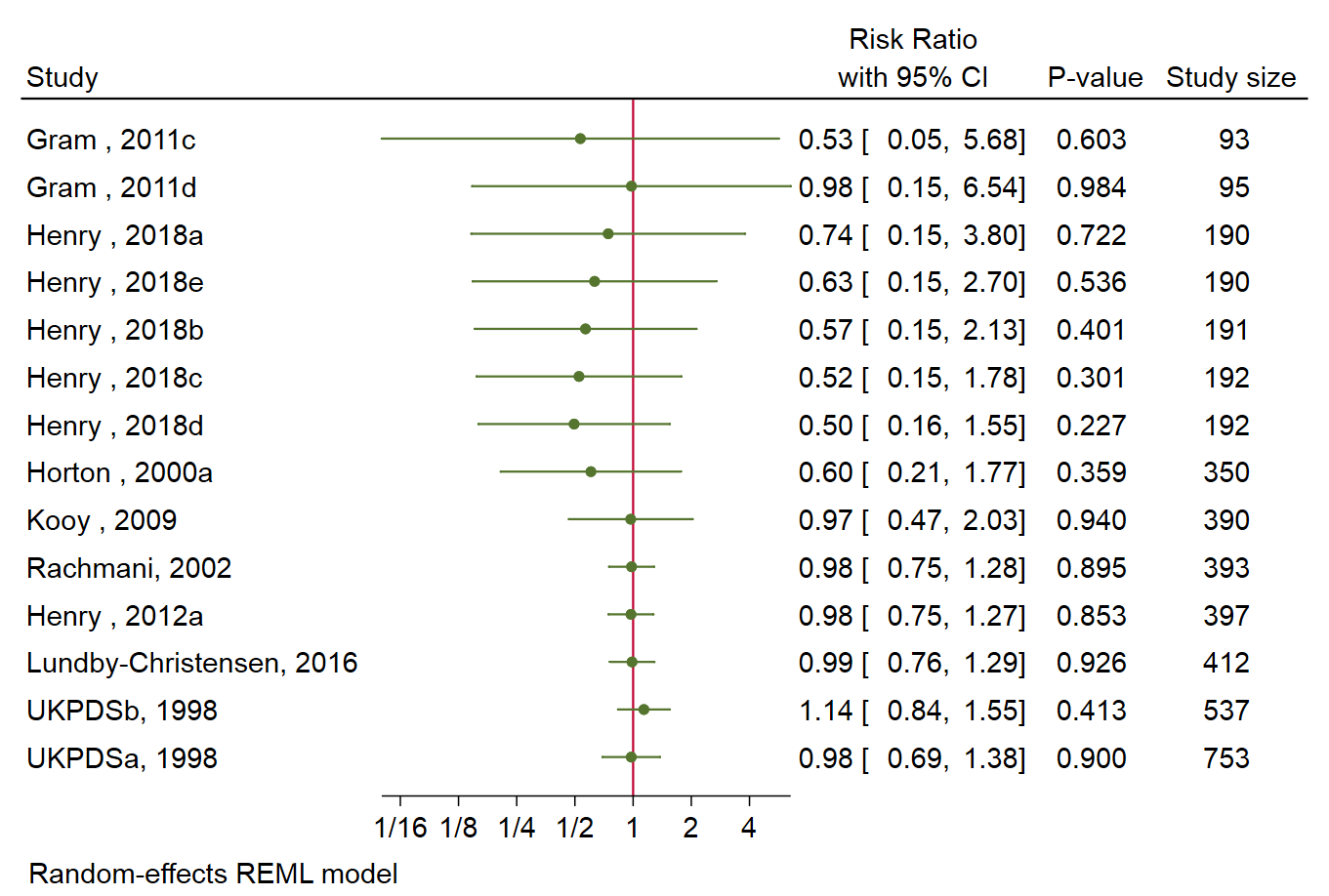


Fig S11. Heterogeneity of all-cause mortality by Labbe plot.


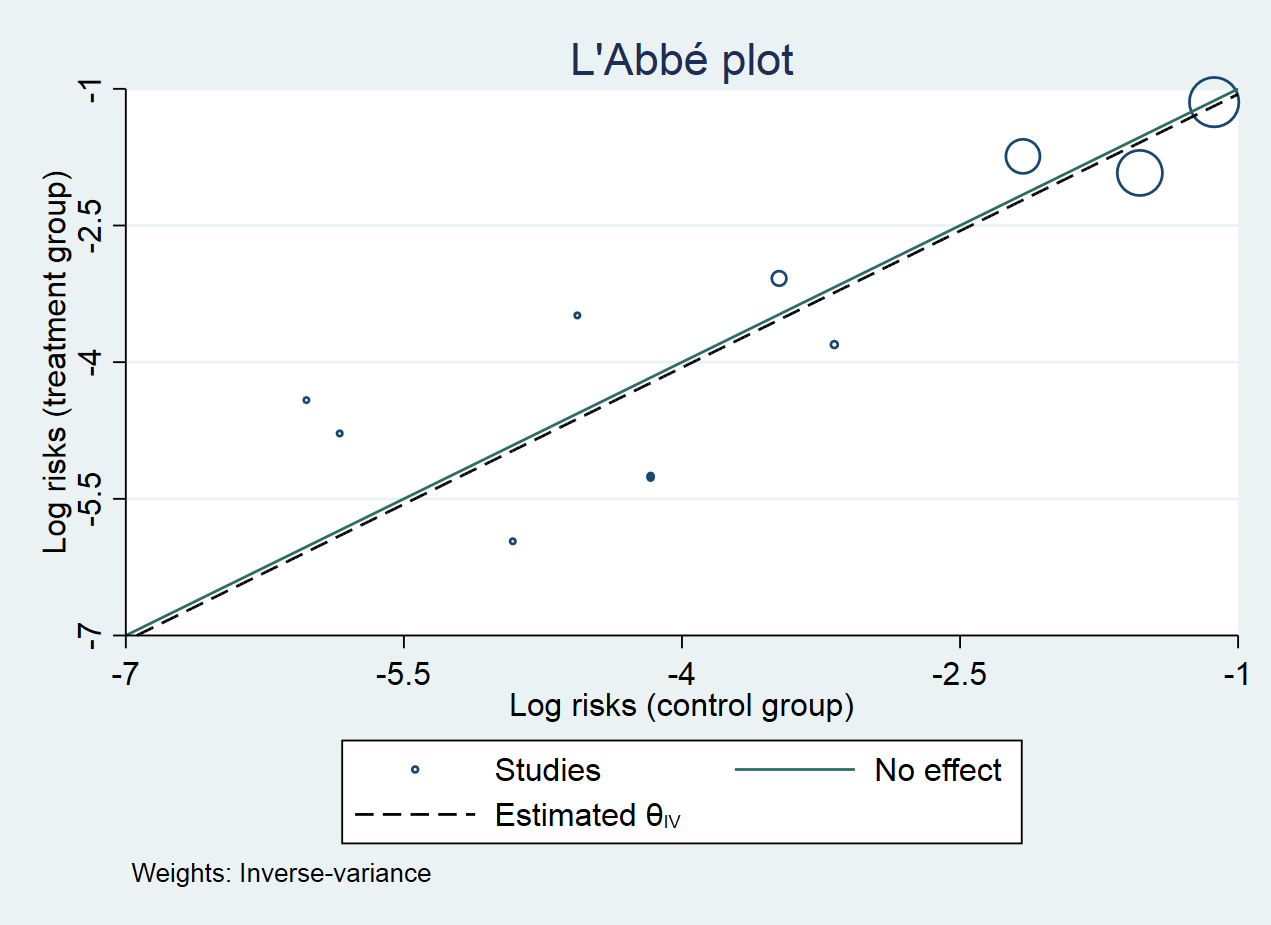


Fig S12. Meta-regression of year of all-cause mortality.


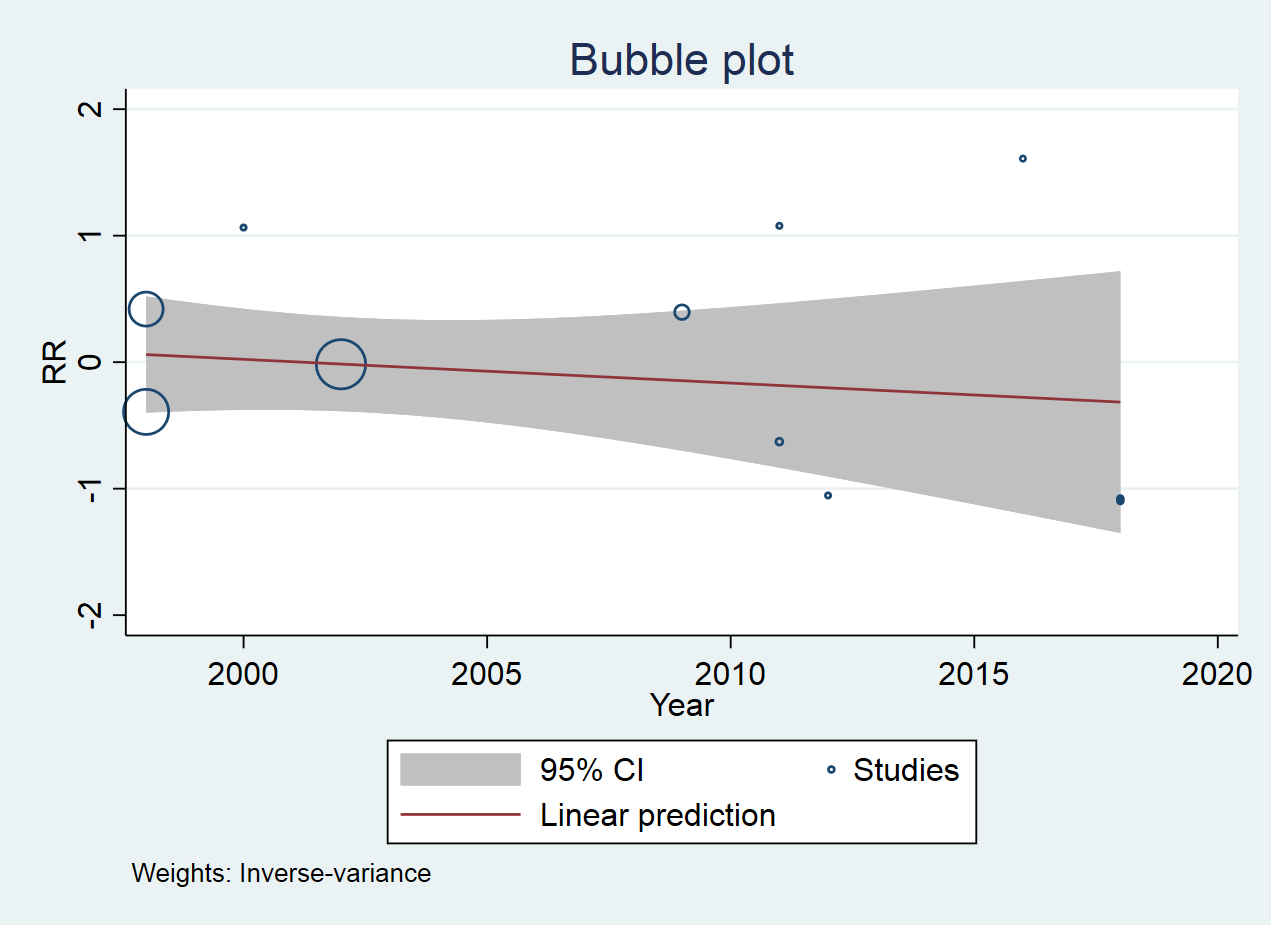


Fig S13. Meta-regression of study size of all-cause mortality.


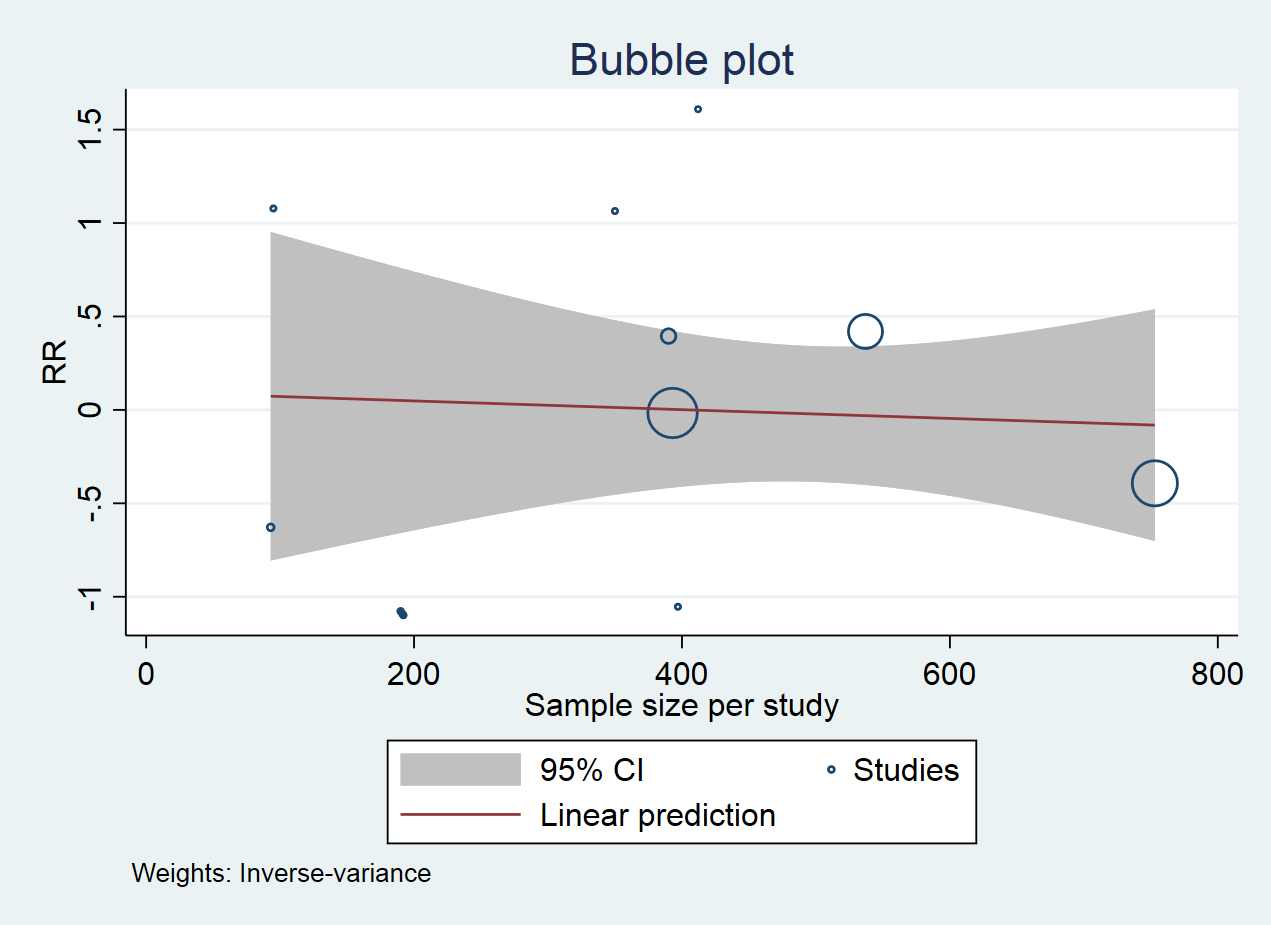


Fig S14. Contour-enhanced funnel plot of all-cause mortality.


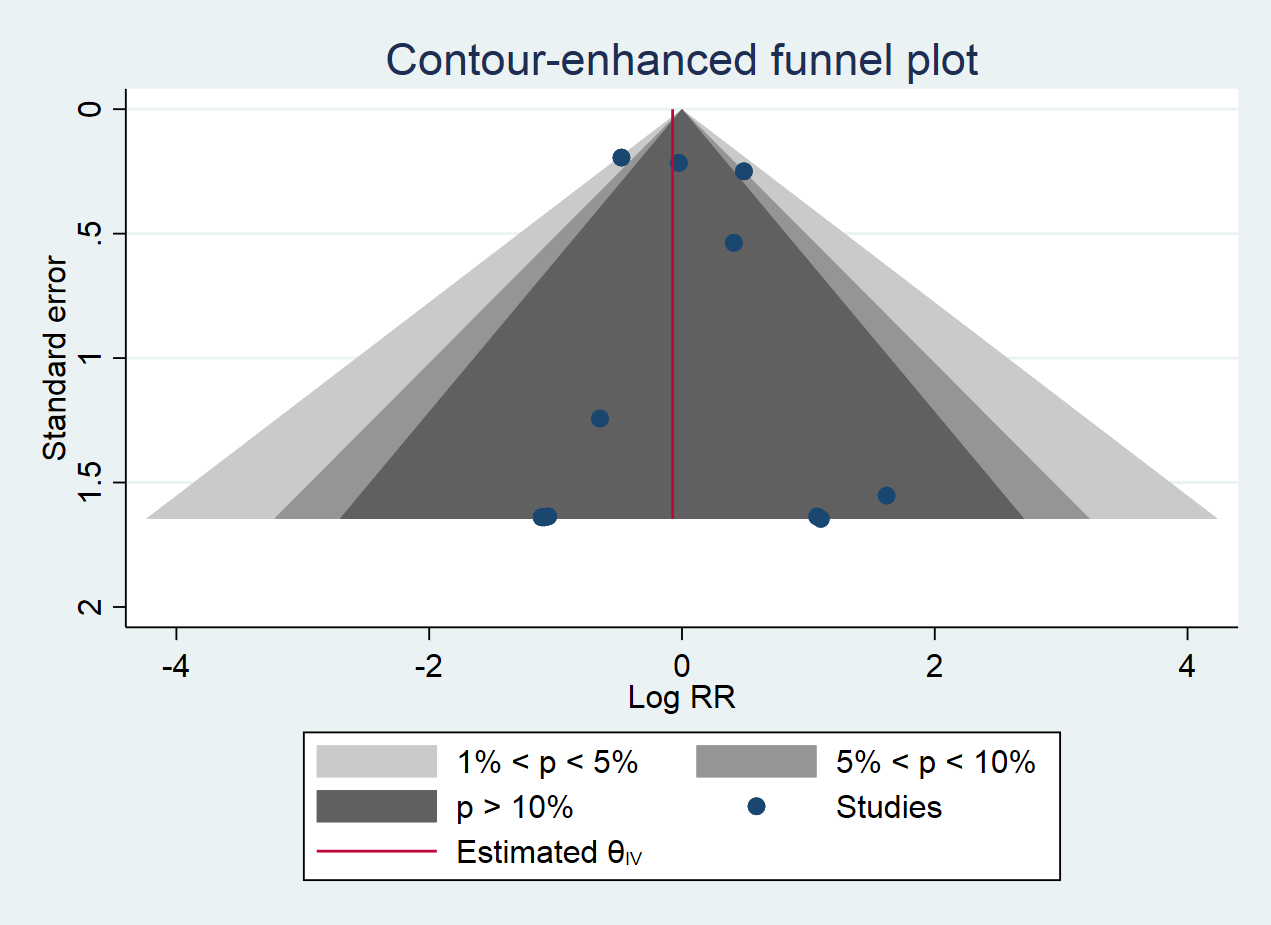


Fig S15. Subgroup analysis of cardiovascular mortality


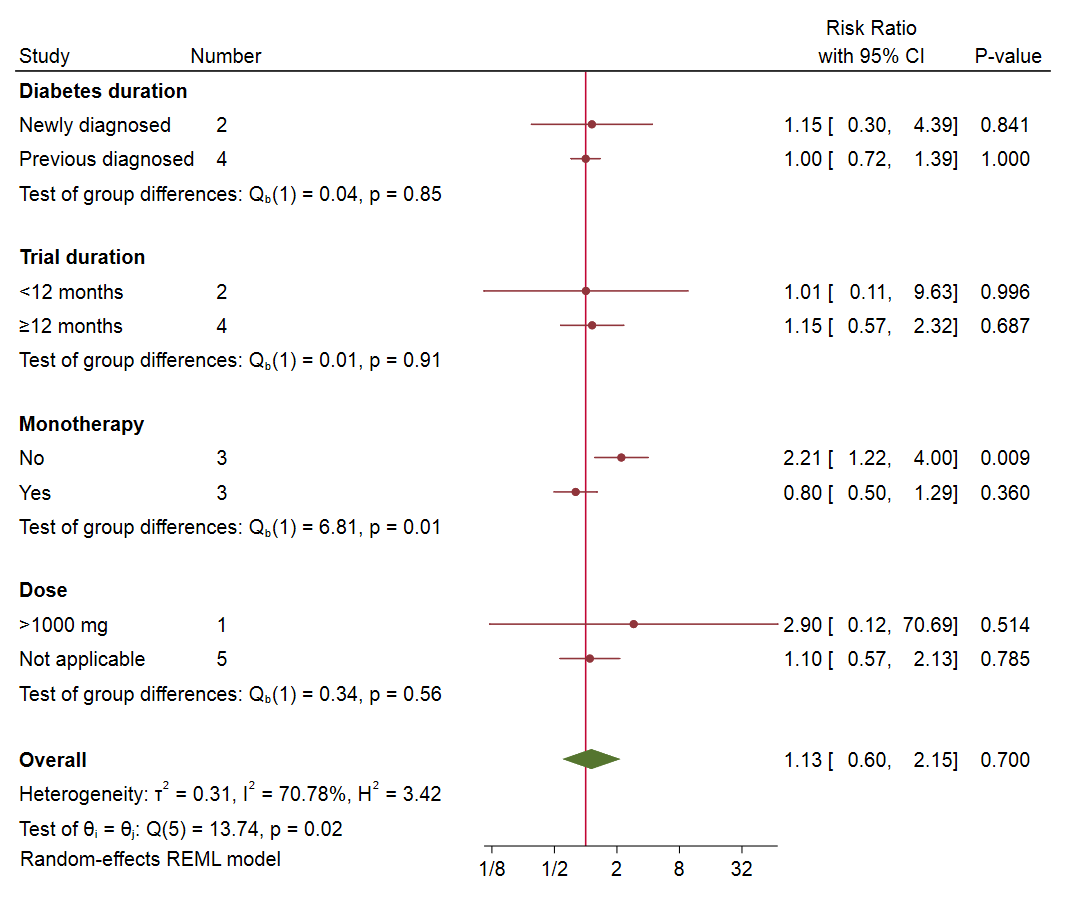


Fig S16. Forest plot of myocardial ischemia


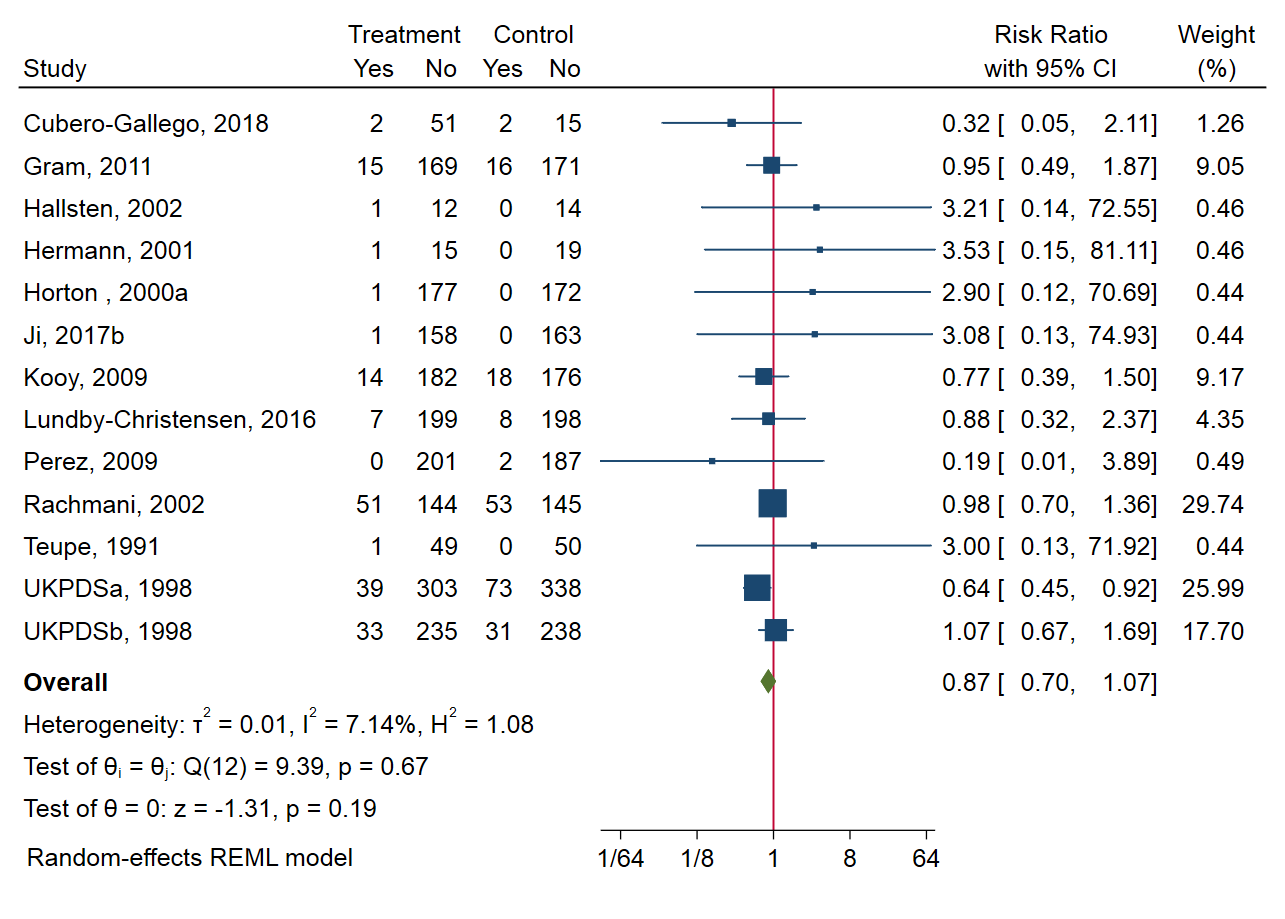


Fig S17. Forest plot of heart failure


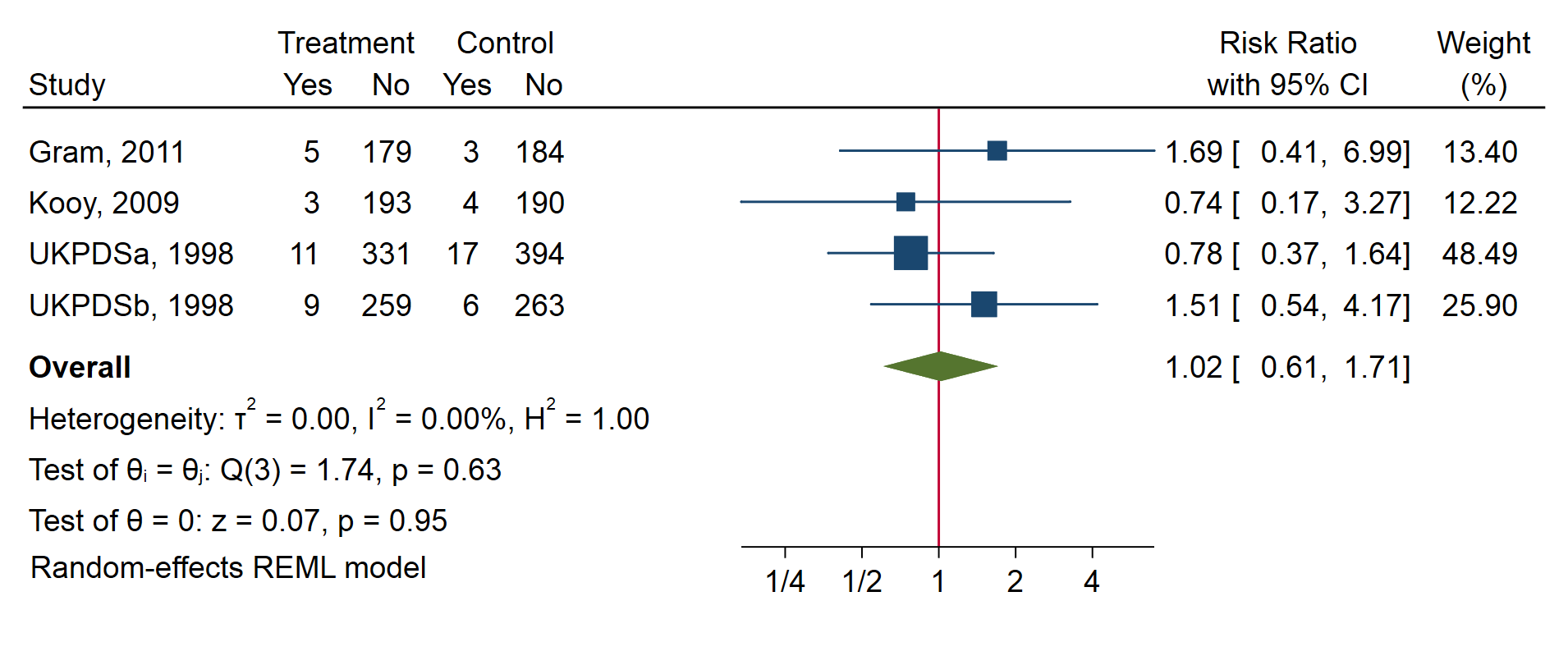


Fig S18. Forest plot of stroke


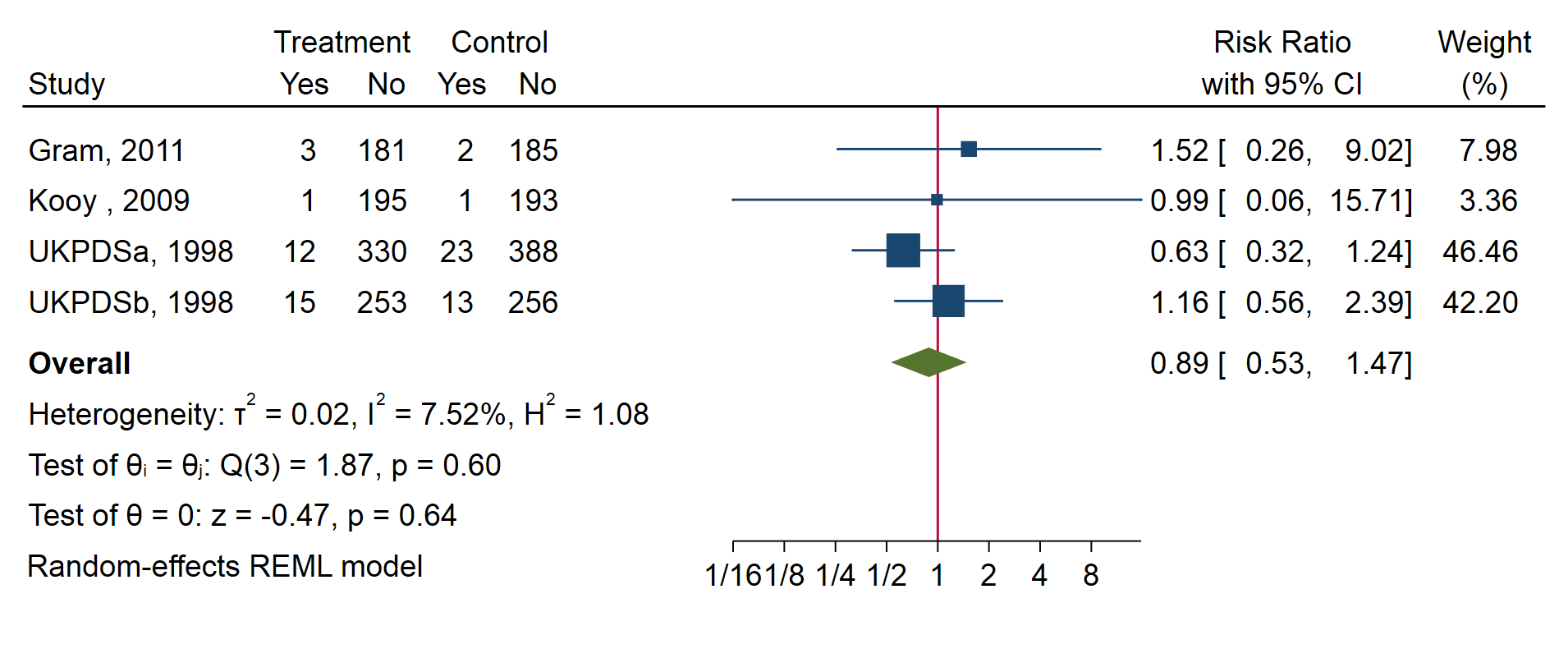


Fig S19. Forest plot of hypertension


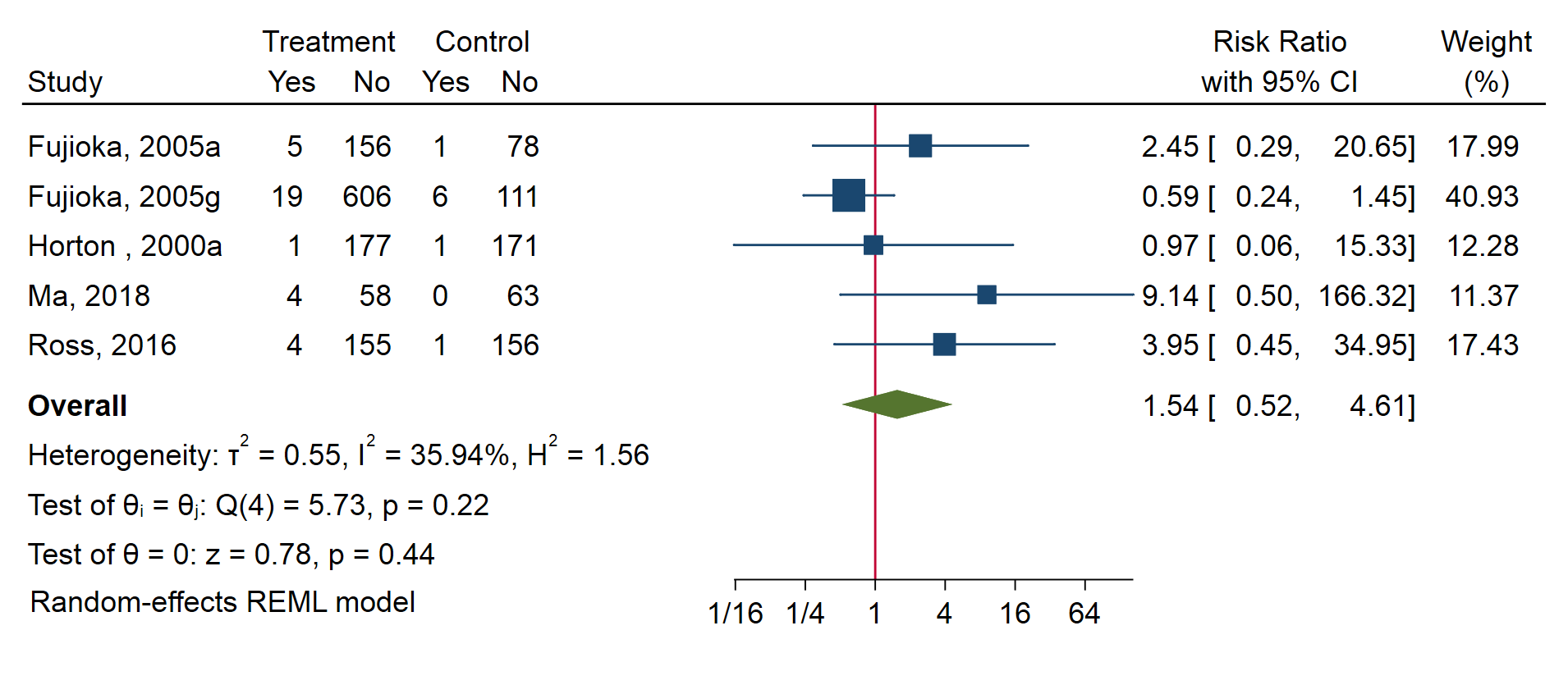


Fig S20. Forest plot of peripheral vascular diseases


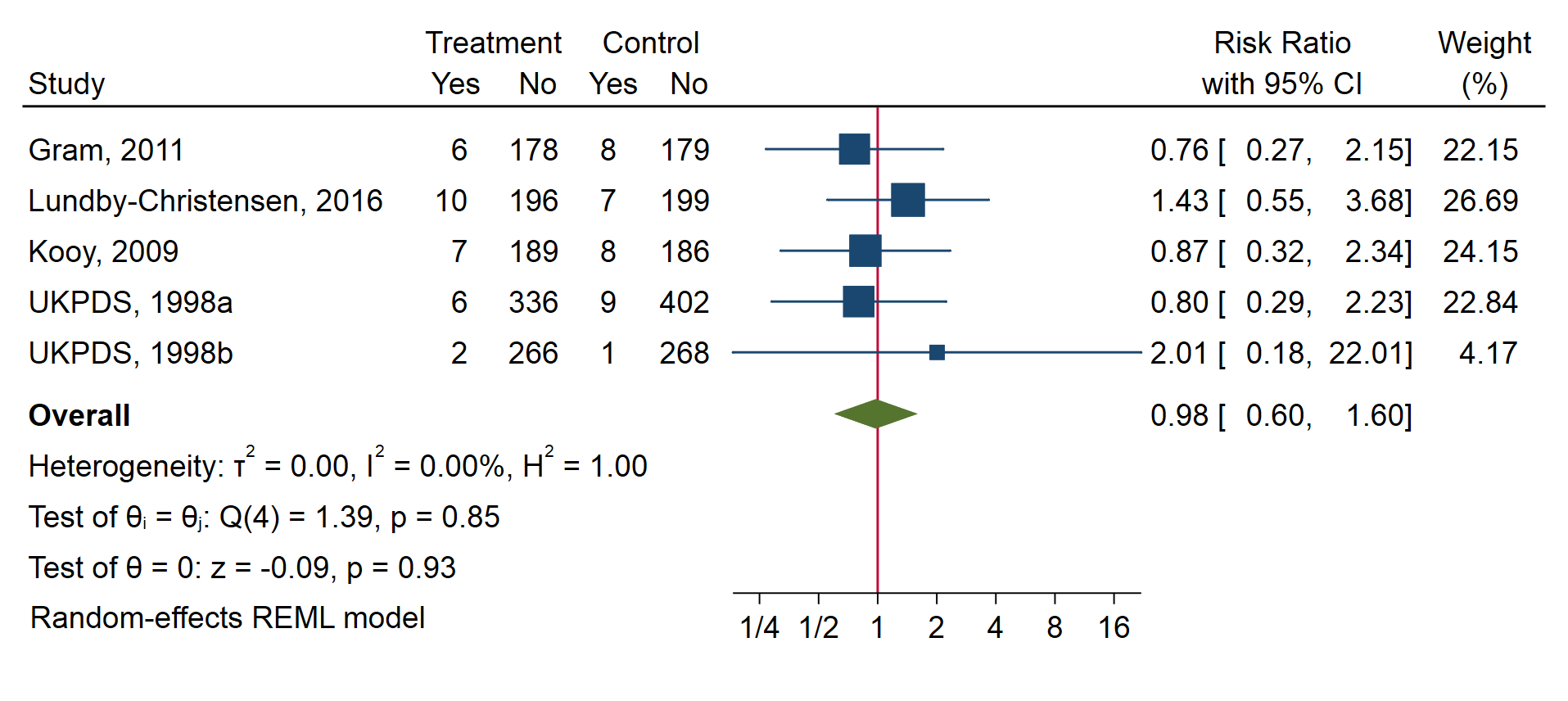


Fig S21. Forest plot of microvascular events


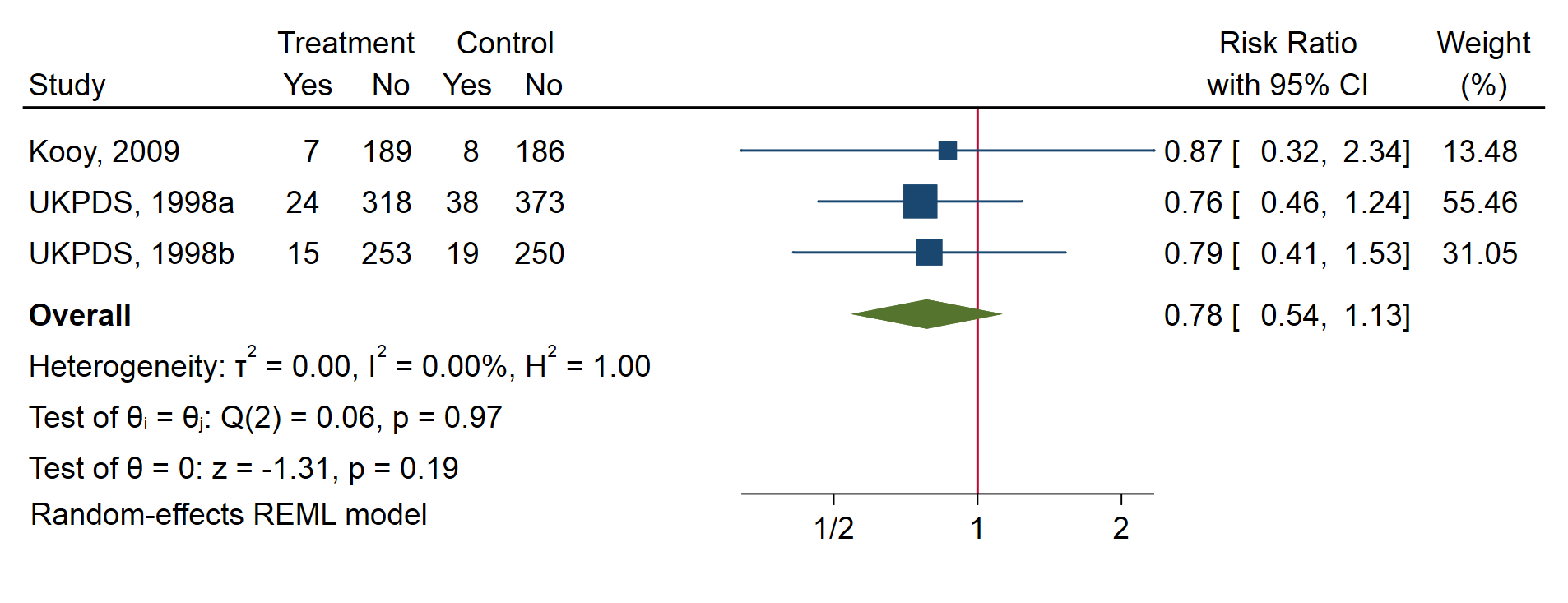


Fig S22. Forest plot of nephropathy


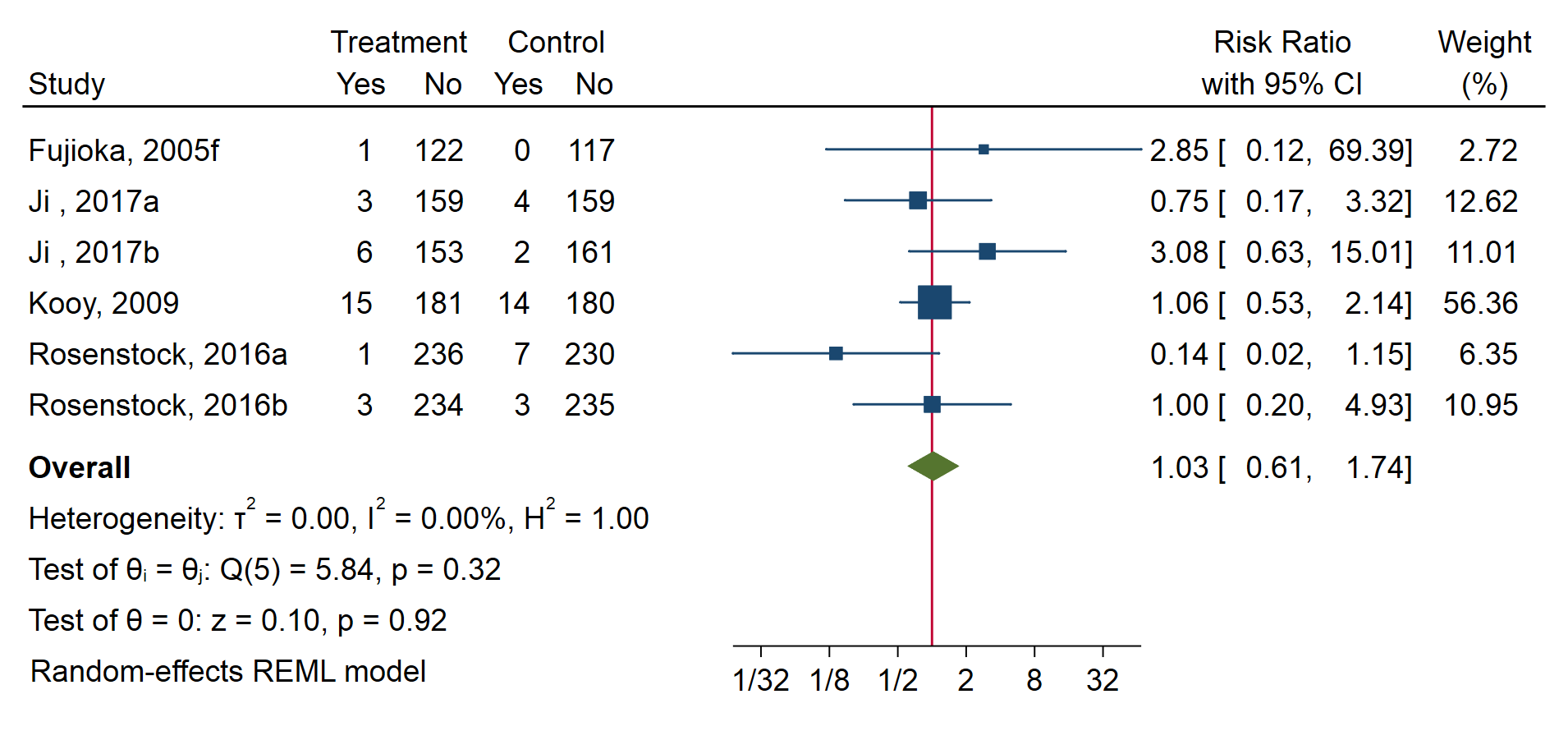


Fig S23. Forest plot of oculopathy


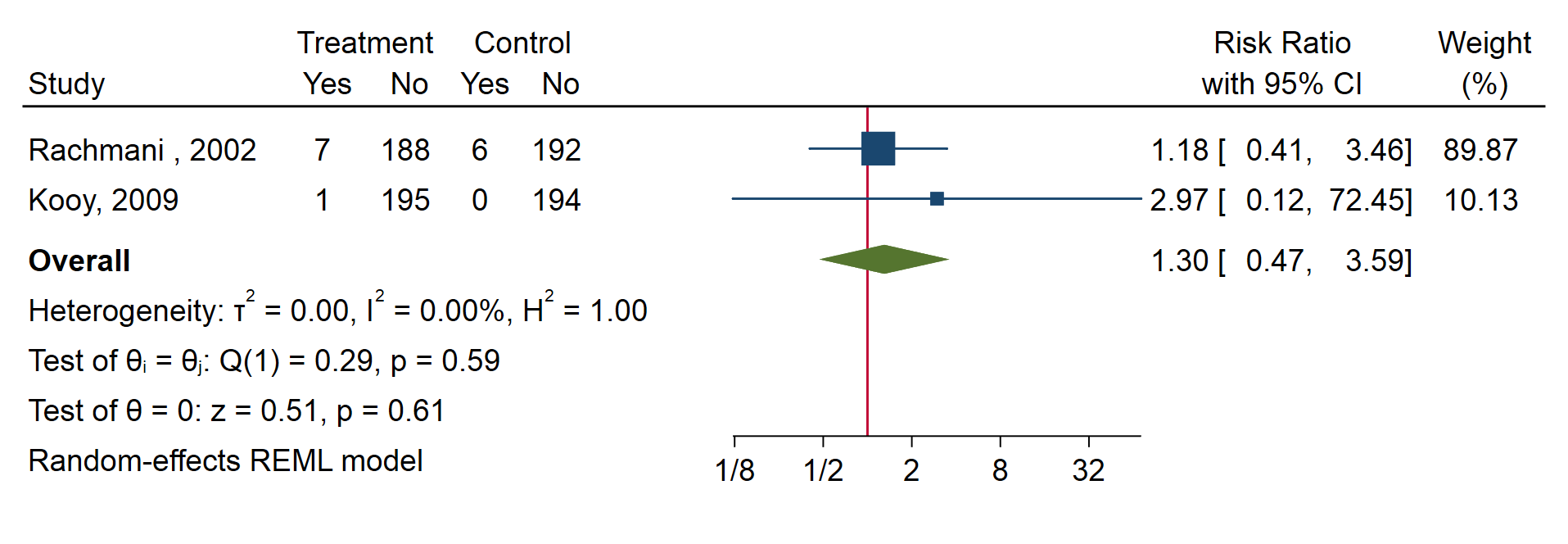


Fig S24. Forest plot of dyslipidemia


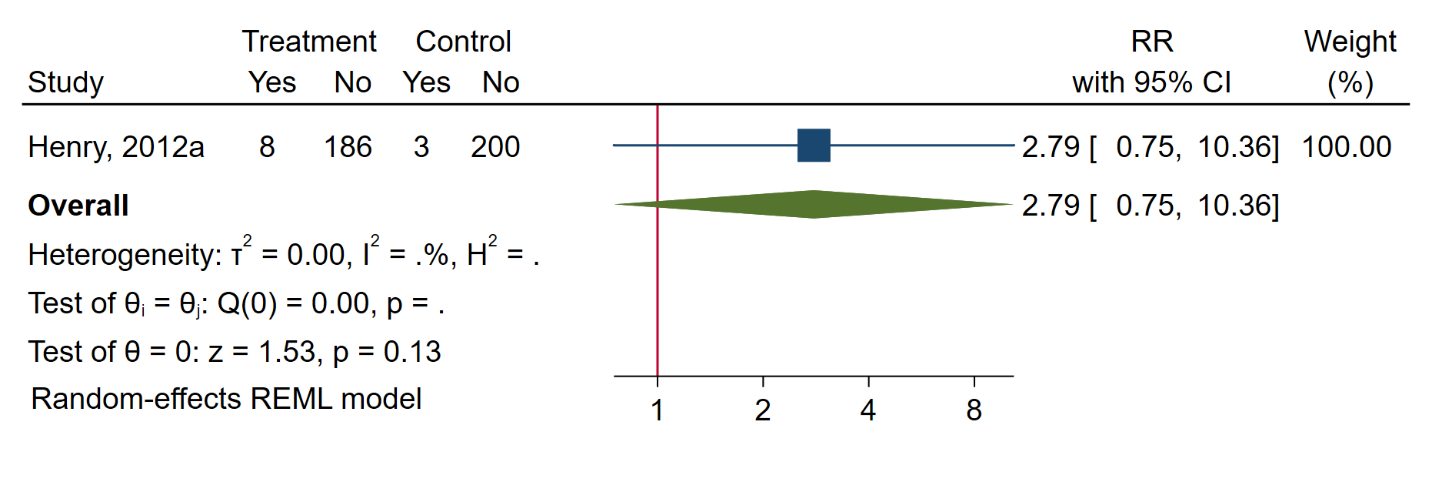


**Table S1. Characteristics of enrolled studies for quantitative analysis.**

| No. | Author/Year | Journal | Ref | Main region | Inter/Con | Metformin Dosage (mg/d) | Number  (Intven/Con) | Sample size | Age (y) | Male (%) | Trail duration (m) | Diabetes duration (y) | HbA1c (%) | Data presentation |
| --- | --- | --- | --- | --- | --- | --- | --- | --- | --- | --- | --- | --- | --- | --- |
|  | Cubero-Gallego  et al 2018 | Rev Esp Cardiol | [20] | Spain | Met/No inter | 2170 | 53/17 | 70 | 66.8 ± 9.5/66.9 ± 9.1 | 80 | 9 | NA | 7.2 ± 0.9/7.9 ± 1.3 | means ± SD |
|  | Fujioka et al 2005a | Diabetes Obes Metab | [21] | USA | Met XR1000/Pla | 1000 | 161/79 | 240 | 55 ± 11/58 ± 11 | 59.6 | 2.8 | 3.3 ± 2.8/3.2 ± 2.6 | 8.1 ± 0.9/7.9 ± 0.9 | means ± SD |
|  | Fujioka et al 2005f | Diabetes Obes Metab | [21] | USA | Met XR1000 bid/Pla | 2000 | 123/117 | 240 | 57 ± 10/54 ± 10 | 48.3 | 3.7 | 3.1 ± 2.7/2.7 ± 2.7 | 8.4 ± 1.1/8.3±1.1 | means ± SD |
|  | Fujioka et al 2005g | Diabetes Obes Metab | [21] | USA | Met XR/Pla | NA | 625/117 | 742 | NA | NA | 3.7 | NA | NA | NA |
|  | Gram et al 2011 | Diabetes Care | [22] | Denmark | See Gram a-d | NA | 184/187 | 371 | 56 | 64.1 | 24 | 8.5 | 8.6 | NA |
|  | Gram et al 2011c | Diabetes Care | [22] | Denmark | Met +Ins Asp/Pla + Ins Asp | NA | 45/48 | 93 | 56.1 ± 8.2/57.1 ± 8.5 | 54.8 | 24 | 8.7 ± 4.5/9.1 ± 5.5 | 8.5 ± 1.2/8.5 ± 1.2 | means ± SD |
|  | Gram et al 2011d | Diabetes Care | [22] | Denmark | Met + Ins Asp + Ros/Ins Asp +Ros | NA | 48/47 | 95 | 55.3 ± 9.1/56.1 ± 8.3 | 64.2 | 24 | 9.0 ± 5.8/9.4 ± 6.3 | 8.5 ± 1.2/8.3 ± 1.0 | means ± SD |
|  | Hallsten et al 2002 | Diabetes | [23] | Finland | Met/Pla | NA | 13/14 | 27 | 57.8 ± 2.2/57.7 ± 1.9 | 66.7 | 6.1 | 0 | 6.9 ± 0.2/6.3 ± 0.1 | means ± SE |
|  | Hermann et al 2001 | Diabetes Obes Metab | [24] | Sweden | Met + Ins/Pla + Ins | NA | 16/19 | 35 | 56.9 ± 10.2/58.1 ± 9.7 | 54.3 | 12 | 13 (3–31)/13 (4–25) | 9.1 ± 1.3/8.7 ± 1.0 | means ± SD  or median (range) |
|  | Henry et al 2012a | Int J Clin Pract | [26] | Euro-America/Asia | Met+ Dap 5 mg/Pla + Dap 5 mg | NA | 194/203 | 397 | 51.7 ± 9.3/52.3 ± 10.2 | 42.8 | 5.6 | 1.6 ± 2.4/1.6 ± 3.1 | 9.2 ± 1.3/9.1 ± 1.4 | means ± SD |
|  | Henry et al 2018a | PLoS One | [25] | USA | Met XR600 mg/Pla | 600 | 94/96 | 190 | 56 ± 10/57 ± 11 | 50.5 | 3.7 | 8.6 ± 0.9/8.6 ± 0.9 | 6.6 ± 5.2/8.3 ± 7.0 | means ± SD |
|  | Henry et al 2018b | PLoS One | [25] | USA | Met XR900 mg/Pla | 900 | 95/96 | 191 | 55 ± 10/57 ± 11 | 55.0 | 3.7 | 8.7 ± 0.8/8.6 ± 0.9 | 8.8 ± 8.0/8.3 ± 7.0 | means ± SD |
|  | Henry et al 2018c | PLoS One | [25] | USA | Met XR1200 mg/Pla | 1200 | 96/96 | 192 | 55 ± 11/57 ± 11 | 51.0 | 3.7 | 8.7 ± 0.9/8.6 ± 0.9 | 7.3 ± 6.3/8.3 ± 7.0 | means ± SD |
|  | Henry et al 2018d | PLoS One | [25] | USA | Met XR1500 mg/Pla | 1500 | 96/96 | 192 | 55 ± 9/57 ± 11 | 52.6 | 3.7 | 8.6 ± 0.9/8.6 ± 0.9 | 7.6 ± 6.0/8.3 ± 7.0 | means ± SD |
|  | Henry et al 2018e | PLoS One | [25] | USA | Met IR2000 mg/Pla | 2000 | 94/96 | 190 | 57 ± 11/57 ± 11 | 51.1 | 3.7 | 8.6 ± 0.9/8.9 ± 0.9 | 8.6 ± 7.1/8.3 ± 7.0 | means ± SD |
|  | Horton et al 2000a | Diabetes Care | [27] | USA | Met/Pla | 1500 | 178/172 | 350 | 56.8 ± 10.9/59.6 ± 10.9 | 64.3 | 5.6 | 4.5 ± 5.5/4.6 ± 4.7 | 8.4 ± 1.2/8.3 ± 1.1 | means ± SD |
|  | Ji et al 2017a | Diabetes Obes Metab | [28] | China/Malaysia/Korea | Met/Pla | 1000 | 162/163 | 325 | 53.6 ± 9.91/52.2 ± 10.17 | 54.5 | 6.1 | NA | 8.40 ± 0.78/8.21 ± 0.77 | means ± SD |
|  | Ji et al 2017b | Diabetes Obes Metab | [28] | China/Malaysia/Korea | Met + Alo/Alo | 1000 | 159/163 | 322 | 53.4 ± 10.46/55.4 ± 9.62 | 58.7 | 6.1 | NA | 8.39 ± 0.81/8.48 ± 0.71 | means ± SD |
|  | Lundby-Christensen  et al 2016 | BMJ Open | [29] | Denmark | Met + Ins/Pla + Ins | 2000 | 206/206 | 412 | 61.0 ± 8.7/60.3 ± 9.1 | 68.2 | 18 | 13.5 ± 6.2/12.2 ± 6.5 | 8.6 ± 1.1/8.5 ± 1.0 | means ± SD |
|  | Ma et al 2018 | J Diabetes Investig | [30] | Asia | Lin +  metformin/Lin | 2000 | 62/63 | 125 | 48.8 ± 10.0/48.6 ± 9.1 | 40.8 | 5.6 | NA | 9.99 ± 1.30/10.06 ± 1.06 | means ± SD |
|  | Kooy et al 2009 | Arch Intern Med | [31] | Netherlands | Met Hyd + Ins/Plac + Ins | NA | 196/194 | 390 | 64 ± 10/59 ± 11 | 45.6 | 51.6 | 14 ± 9/12 ± 8 | 7.9 ± 1.2/7.9 ± 1.2 | means ± SD |
|  | Perez et al 2009 | Curr Med Res Opin | [32] | NA | Met + Pioglitazone/ Pioglitazone | 1700 | 201/189 | 390 | 54.7 ± 12.2/54.0 ± 12.1 | 38.5 | 5.6 | NA | 8.89/8.69 | means |
|  | Rachmani et al 2002 | Eur J Intern Med | [33] | Israel | Met/Diet | NA | 195/198 | 393 | 65 ± 4/64 ± 4 | 52.2 | 48 | 15 ± 3/14 ± 4 | 8.6 ± 0.5/8.6 ± 0.4 | means ± SE |
|  | Rosenstock 2016a | Diabetes Care | [34] | USA | Met + CANA100/CANA100 | NA | 237/237 | 474 | 54.2 ± 9.6/54.0 ± 10.7 | 44.9 | 7 | 2.9 ± 3.3/3.5 ± 4.4 | 8.8 ± 1.1/8.8 ± 1.2 | means ± SD |
|  | Rosenstock 2016b | Diabetes Care | [34] | USA | Met + CANA300/CANA300 | NA | 237/238 | 475 | 55.4 ± 9.8/55.8 ± 9.6 | 50.5 | 7 | 3.3 ± 3.9/3.3 ± 4.4 | 8.9 ± 1.2/8.8 ± 1.2 | means ± SD |
|  | Ross et al 2016 | Postgrad Med | [35] | Canada | Met + Lin/Lin | 2000 | 159/157 | 316 | 49.0 ± 10.9/48.6 ± 11.2 | 46.2 | 5.6 | 0 | 9.79 ± 1.19/9.88 ± 1.10 | means ± SD |
|  | Teupe et al 1991 | Diabete Metab | [36] | Germany | Met + Diet/Diet | 1700 | 50/50 | 100 | 51.5±10.1/56 ±7.6 | 40 | 24 | 8.1±6.7/6.4 ±4.9 | 10.0 ± 1.6/9.6 ± 1.3 | means ± SD |
|  | UKPDS Group 1998a | Lancet | [37] | UK | Met + Diet/Diet | NA | 342/411 | 753 | 53 ± 8/53 ± 9 | 46.5 | 128.4 | NA | 7.3 ± 1.5/7.1 ± 1.5 | means ± SD |
|  | UKPDS Group 1998b | Lancet | [37] | UK | Met + Sul/Sul | NA | 268/269 | 537 | 59 ± 8/58 ± 9 | 60.0 | 79.2 | NA | 7.5 ± 1.7/7.6 ± 1.8 | means ± SD |

**Abbreviations**: Met, metformin; Pla, placebo; Ref, reference; No inter, no intervention; XR, XR, sustained release tablet; Ins, insulin; IR, immediate release; bid, twice a day; Ins Asp, insulin aspart; Ros, rosiglitazone; Hyd, hydrochloride; Dap, dapagliflozin; Alo, alogliptin; Lin, linagliptin; Hyd, hydrochloride; CANA, canagliflozin; Sul, sulfonylurea; y, year; m, month

**Table S2. Characteristics of enrolled studies for qualitative analysis**

| No. | Author/Year | Journal | Reference | Main region | Intven/Con | Number (Intven/Con) | Sample size | Age (y) | Male (%) | Trail duration (m) | Diabetes duration (y) | HbA1c (%) | Data presentation |
| --- | --- | --- | --- | --- | --- | --- | --- | --- | --- | --- | --- | --- | --- |
|  | Boule et al 2011 | Diabetes Care | [38] | Canada | Met/Pla | 10/10 | 20 | 58 ± 6 | 80 | 0.9 | NA | 6.5±0.6 | means ± SD |
|  | Chiasson et al 2001a | Diabetes Care | [39] | Canada | Met/Pla | 83/83 | 166 | 57.9 ± 8.6/57.7 ± 9.9 | 70.5 | 8.4 | 7.5±7.4/5.1±4.9 | 8.2±0.9/8.1±0.7 | means±SD |
|  | Chiasson et al 2001b | Diabetes Care | [39] | Canada | Met + Mig/Mig | 76/82 | 158 | 58.9 ± 7.9/57.3 ± 9.0 | 77.8 | 8.4 | 6.1±5.5/5.2±4.7 | 8.3±0.8/8.2±0.9 | means±SD |
|  | Chien et al 2007 | J Chin Med Assoc | [40] | China | Met + Gly/Gly | 21/17 | 38 | 57 ± 8/63 ± 7 | 57.9 | 3.7 | 6.6±5.6/8.6±5.9 | 8.85±1.21/8.69±0.94 | means ± SD |
|  | DeFronzo et al 1995a | N Engl J Med | [41] | USA | Met/Pla | 143/146 | 289 | 53 ± 1/53 ± 1 | 42.9 | 6.8 | 6.0±0.5/6.0±0.6 | 8.4±0.1/8.2±0.2 | means ± SE |
|  | DeFronzo et al 1995b | N Engl J Med | [41] | USA | Met + Gly/Gly + Pla | 213/209 | 422 | 55 ± 1/56 ± 1 | 46.0 | 6.8 | 7.8±0.4/8.7±0.4 | 8.8±0.1/8.5±0.1 | means ± SE |
|  | Eurich et al 2009 | Trials | [42] | Canada | Met/Pla | 27/26 | 53 | 74.9 ± 8.7/78.2 ± 8.5 | 56.6 | 6 | NA | 7.7±2.6/6.8±1.2 | means ± SD |
|  | Fujioka et al 2005b | Diabetes Obes Metab | [21] | USA | Met XR500/Pla | 128/117 | 245 | 55 ± 11/54 ± 10 | 46.1 | 3.7 | 3.3±2.9/2.7±2.7 | 8.2±0.9/8.3±1.1 | means ± SD |
|  | Fujioka et al 2005c | Diabetes Obes Metab | [21] | USA | Met XR1000/Pla | 120/117 | 237 | 56 ± 10/54 ± 10 | 53.6 | 3.7 | 3.0±2.7/2.7±2.7 | 8.4±1.1/8.3±1.1 | means ± SD |
|  | Fujioka et al 2005d | Diabetes Obes Metab | [21] | USA | Met XR1500/Pla | 120/117 | 237 | 56 ± 11/54 ± 10 | 48.5 | 3.7 | 2.9±2.7/2.7±2.7 | 8.4±1.0/8.3±1.1 | means ± SD |
|  | Fujioka et al 2005e | Diabetes Obes Metab | [21] | USA | Met XR2000/Pla | 134/117 | 251 | 55 ± 11/54 ± 10 | 44.2 | 3.7 | 2.7±2.5/2.7±2.7 | 8.4±1.1/8.3±1.1 | means ± SD |
|  | Gram et al 2011a | Diabetes Care | [22] | Denmark | Met + NPH ins/Pla + NPH ins | 45/46 | 91 | 55.4 ± 8.5/55.8 ± 7.7 | 64.8 | 24 | 8.2 ± 4.0/7.3 ± 4.3 | 8.9 ± 1.2/8.7 ± 1.3 | means ± SD |
|  | Gram et al 2011b | Diabetes Care | [22] | Denmark | Met + Ros +  NPH Ins/Ros +  NPH Ins | 46/46 | 92 | 57.3 ± 8.2/57.3 ± 8.9 | 63.0 | 24 | 8.1 ± 5.3/9.2 ± 6.9 | 8.5 ± 1.1/8.7 ± 1.2 | means ± SD |
|  | Grant et al 1996a | Diabetes Care | [43] | UK | Met 1500 mg/Pla | 25/23 | 48 | NA | NA | 5.6 | NA | NA | NA |
|  | Grant et al 1996b | Diabetes Care | [43] | UK | Met 3000 mg/Pla | 27/23 | 50 | NA | NA | 5.6 | NA | NA | NA |
|  | Henry et al 2012b | Int J Clin Pract | [26] | Euro-America/Asia | Me + Dap/Pla+ Dap | 211/219 | 430 | 2.2 ± 3.3/2.1 ± 3.8 | 49.1 | 5.6 | 51.0±10.1/51.1±11.5 | 9.1±1.3/9.1±1.3 | means ± SD |
|  | Horton et al 2000b | Diabetes Care | [27] | USA | Met + Nat/Nat | 172/179 | 351 | 58.4 ± 10.9/58.6 ± 10.7 | 60.1 | 5.6 | 4.5±5.3/4.7±5.5 | 8.4±1.1/8.3±1.0 | means ± SD |
|  | Horton et al 2004 | Curr Med Res Opin | [44] | USA | Met/Pla | 104/104 | 208 | 55.4 ± 1.1/59.0 ± 1.1 | 65.9 | 5.6 | 3.7±0.4/4.2±0.4 | 8.3±0.1/8.2±0.1 | NA |
|  | Kovacs et al 2014a | Diabetes Obes Metab | [45] | Euro-America/Asia | Met + Pig + Pla/Pig + Pla | 124/41 | 165 | 54.6 ± 10.5 | 44.2 | 5.6 | NA | 8.2± 0.92 | means ± SD |
|  | Kovacs et al 2014b | Diabetes Obes Metab | [45] | Euro-America/Asia | Met+ Pig + Emp 10 mg/Pig + Emp 10 mg | 125/40 | 165 | 54.7 ± 9.9 | 50.3 | 5.6 | NA | 8.1 ± 0.89 | means ± SD |
|  | Kovacs et al 2014c | Diabetes Obes Metab | [45] | Euro-America/Asia | Met + Pig + Emp 20 mg/Pig + Emp 20 mg | 127/41 | 168 | 54.2 ± 8.9 | 50.6 | 5.6 | NA | 8.1 ± 0.82 | means ± SD |
|  | Lim et al 2017 | Diabetes Obes Metab | [46] | Korea/Thailand | Met + Gem/Gem +Pla | 136/140 | 276 | 54.4 ± 10.4/53.4 ± 11.0 | 57.2 | 5.6 | 4.2±4.3/3.5±4.6 | 8.65±0.88/8.66±0.90 | means ± SD |
|  | Manzella et al 2004 | Am J Hypertens | [47] | Italy | Met + Diet/Pla + Diet | 60/60 | 120 | 57 ± 11 | 53.3 | 4 | NA | 8.0±0.2/8.1±0.2 | means ± SD |
|  | Nar et al 2009 | Acta Diabetol | [48] | Turkey | Met/Lif | 19/15 | 34 | 49.4 ± 8.6/44.5 ± 5.9 | 26.5 | 6 | 0 | 6.9±1.4/6.1±1.1 | means ± SE |
|  | Natali et al 2004 | Diabetes Care | [49] | Italy/UK | Met/Pla | 28/22 | 50 | 58 ± 10/58 ± 9 | 80 | 3.7 | 6.3±5.3/3.4±3.4 | 7.8±1.1/7.6±0.8 | means ± SD |
|  | Srivastava  et al 2017a | J Am Heart Assoc | [50] | USA | Met/Pla | 107/102 | 209 | 55.0 (47.0–64.0)/  55.0 (46.0–62.0) | 47.8 | 2.8 | 1.0 (0.2–6.0)/  1.8 (0.2–5.4) | 6.7 (6.3–7.5)/  6.9 (6.4–7.8) | median (interquartile range) |
|  | Srivastava et al 2017b | J Am Heart Assoc | [50] | USA | Met+Ins/Pla+Ins | 117/112 | 229 | 55.0 (48.0–62.0)/  53.0 (46.0–63.0) | 41.0 | 2.8 | 2.0 (0.3–5.2) /  2.7 (0.4–5.9) | 7.1 (6.4–7.8)/  6.9 (6.3–7.5) | median (interquartile range) |
|  | Sullivan et al 2011 | Diabetes Res Clin Pract | [51] | Australia | Met + Pla + Fen/Diet + Pla + Fen | 868/1327 | 2195 | NA | NA | 60 | NA | NA | NA |
|  | Uehara 2001 | Diabetes Obes Metab | [52] | Brazil | Met/Pla | 11/11 | 22 | 57.2 ± 4.3/57.5 ± 6.7 | 27.3 | 2.8 | 3/3.7 | 5.3±1.5/6.7±3.0 | means ± SD |
|  | Wulffele 2004 | J Intern Med | [53] | Netherlands | Met/Pla | 171/182 | 353 | 63.2 ± 9.8/58.9 ± 11.1 | 47.3 | 3.7 | 14.0±8.4/12.0±8.0 | 7.9±1.2/7.9±1.2 | means±SD |

**Abbreviations:** Met, metformin; Pla, placebo; Ref, reference; Mig, miglitol; Gly, glyburide; XR, sustained release tablet; Ins, insulin; NPH, neutral protamine hagedorn; Ros, rosiglitazone; Dap, dapagliflozinp; Nat, nateglinide; Pig, pioglitazone; Emp, empagliflozin;

Gem, gemigliptin; Lif, lifestyle changes; Fen, fenofibrate. y, year; m, month

**Table S3. Search strategy**

| Database | No | PICOS | Strategy | Results |
| --- | --- | --- | --- | --- |
| PubMed | #1 | P | Diabetes Mellitus [MeSH] OR Type 2 Diabetes Mellitus OR Diabetes OR Type 2 Diabetes OR Diabetes Mellitus OR Diabetic | 745910 |
|  | #2 | I | Metformin[MeSH] OR Dimethylbiguanidine OR Dimethylguanylguanidine OR Metformin | 21612 |
|  | #3 | C | NA | NA |
|  | #4 | O | Cardiovascular Diseases[MeSH] OR Diabetic Nephropathies[MeSH] OR Diabetic Retinopathy[MeSH] OR Peripheral Vascular Diseases[MeSH] OR heart OR cardiovascular OR myocardial OR stroke OR hypertension OR coronary heart disease OR coronary heart diseases OR mortality OR ischemia OR ischemic OR peripheral vascular disease OR peripheral vascular diseases OR atherosclerosis OR Kidney Diseases OR Kidney Disease OR Retinal Disease OR Retinal Diseases OR Peripheral Vascular Disease | 5235554 |
|  | #5 | S | (randomized controlled trial[Publication Type] OR randomized[TIAB] OR randomised[TIAB] OR placebo[TIAB])) NOT (Review[Publication Type]) NOT (meta-analysis[Publication Type]) NOT (Comment[Publication Type]) NOT (Letter[Publication Type]) | 723941 |
|  | #6 = #1AND#2 AND#4 AND #5 | NA | ((((Diabetes Mellitus [MeSH] OR Type 2 Diabetes Mellitus OR Diabetes OR Type 2 Diabetes OR Diabetes Mellitus OR Diabetic)) AND (Metformin[MeSH] OR Dimethylbiguanidine OR Dimethylguanylguanidine OR Metformin)) AND (Cardiovascular Diseases[MeSH] OR Diabetic Nephropathies[MeSH] OR Diabetic Retinopathy[MeSH] OR Peripheral Vascular Diseases[MeSH] OR heart OR cardiovascular OR myocardial OR stroke OR hypertension OR coronary heart disease OR coronary heart diseases OR mortality OR ischemia OR ischemic OR peripheral vascular disease OR peripheral vascular diseases OR atherosclerosis OR Kidney Diseases OR Kidney Disease OR Retinal Disease OR Retinal Diseases OR Peripheral Vascular Disease)) AND ((randomized controlled trial[Publication Type] OR randomized[TIAB] OR randomised[TIAB] OR placebo[TIAB])) NOT (Review[Publication Type]) NOT (meta-analysis[Publication Type]) NOT (Comment[Publication Type]) NOT (Letter[Publication Type])) | 829 |
| Embase | #7 | P | 'diabetes mellitus'/exp OR 'type 2 diabetes mellitus':ab,ti OR 'type 2 diabetes':ab,ti OR ‘Diabetic’:ab,ti OR ‘Diabetes’:ab,ti | 1135846 |
|  | #8 | I | 'metformin'/exp OR 'dimethylbiguanidine':ab,ti OR 'dimethylguanylguanidine':ab,ti OR ‘metformin’:ab,ti | 64420 |
|  | #9 | C | NA | NA |
|  | #10 | O | 'cardiovascular disease'/exp OR 'diabetic nephropathy'/exp OR 'diabetic retinopathy'/exp OR 'peripheral vascular disease'/exp OR ‘heart’:ab,ti OR ‘cardiovascular’:ab,ti OR ‘myocardial’:ab,ti OR ‘stroke’:ab,ti OR ‘hypertension’:ab,ti OR ‘coronary’:ab,ti OR ‘mortality’:ab,ti OR ‘ischemia’:ab,ti OR ‘ischemic’:ab,ti OR ‘peripheral vascular disease’:ab,ti OR ‘peripheral vascular diseases’:ab,ti OR ‘atherosclerosis’:ab,ti OR ‘atherosclerotic’:ab,ti OR ‘Kidney Diseases’:ab,ti OR ‘Kidney Disease’:ab,ti OR ‘Retinal Disease’:ab,ti OR ‘Retinal Diseases’:ab,ti OR ‘Peripheral Vascular Disease’:ab,ti | 5805774 |
|  | #11 | S | 'randomized controlled trial'/exp NOT review:it | 575354 |
|  | #12 = #7 AND #8 AND #10 AND #11 | NA | ('diabetes mellitus'/exp OR 'type 2 diabetes mellitus':ab,ti OR 'type 2 diabetes':ab,ti OR ‘Diabetic’:ab,ti OR ‘Diabetes’:ab,ti) AND ('metformin'/exp OR 'dimethylbiguanidine':ab,ti OR 'dimethylguanylguanidine':ab,ti OR ‘metformin’:ab,ti) AND ('cardiovascular disease'/exp OR 'diabetic nephropathy'/exp OR 'diabetic retinopathy'/exp OR 'peripheral vascular disease'/exp OR ‘heart’:ab,ti OR ‘cardiovascular’:ab,ti OR ‘myocardial’:ab,ti OR ‘stroke’:ab,ti OR ‘hypertension’:ab,ti OR ‘coronary’:ab,ti OR ‘mortality’:ab,ti OR ‘ischemia’:ab,ti OR ‘ischemic’:ab,ti OR ‘peripheral vascular disease’:ab,ti OR ‘peripheral vascular diseases’:ab,ti OR ‘atherosclerosis’:ab,ti OR ‘atherosclerotic’:ab,ti OR ‘Kidney Diseases’:ab,ti OR ‘Kidney Disease’:ab,ti OR ‘Retinal Disease’:ab,ti OR ‘Retinal Diseases’:ab,ti OR ‘Peripheral Vascular Disease’:ab,ti) AND ('randomized controlled trial'/exp NOT review:it) | 1589 |

**Table S4. Inclusion/exclusion criteria of literature**

| PICOS | Inclusion | Exclusion |
| --- | --- | --- |
| P | 1) Adults (≥18 y) with type 2 diabetes, irrespective of age, gender, and race | Children, pregnant women, and people with only prediabetic state, impaired fasting glucose, or insulin resistance |
| I | 1) Any dose and preparation of oral metformin/metformin analogue (metformin hydrochloride, metformin sustained release tablet, Glucophage, et al)  2) ) Combination of any dose and preparation of oral metformin/metformin analogue (metformin hydrochloride, Glucophage, et al) and antidiabetics  3) No limit on sample size | 1) Trial duration is less than 4 weeks  2) Method of metformin administration is inconsistent between the trial group and the control |
| C | 1) No intervention, or with placebo or a lifestyle intervention  2) The same antidiabetics | Comparing antidiabetics A with antidiabetics B (not including metformin) |
| O | Reporting mortality, cardiac, macrovascular, microvascular, or cardio-metabolic outcomes (cardiovascular mortality, myocardial infarction, stroke, heart failure, ischemic heart disease, hypertension, nephropathy, oculopathy, peripheral vascular diseases) | Not available |
| S | RCT irrespective of blinding or arm | 1) Articles without peer-reviewed or unpublished  2) Studies that were repeatedly published or had qualitative outcomes  3) Quasi-experimental studies, crossover, and observational studies |

**Table S5. Reasons for excluded literature**

| Exclusion | Reason | Number | Description |
| --- | --- | --- | --- |
|  | Case report | 1 |  |
|  | Case-control study | 1 |  |
|  | Cohort study | 8 |  |
|  | Prediabetes | 58 | Including impaired fasting glucose，impaired glucose tolerance, and insulin resistance |
|  | Non-diabetes | 47 |  |
|  | Review/comment | 28 |  |
| Excluded abstract | systematic review/meta-analysis | 38 |  |
|  | T1DM | 24 | Type 1 diabetes mellitus |
|  | irrelevant theme | 1701 |  |
|  | Children | 38 | <18 years |
|  | Conference abstract | 5 |  |
|  | Basic research | 11 |  |
|  | Pregnancy | 31 |  |
|  | Subtotal | 1991 |  |
|  | No blank control | 63 | Comparing metformin monotherapy with another durg or comparing antidiabetics A with antidiabetics B |
|  | No data on outcome  of interest | 5 |  |
| Excluded full text | Trial duration < 28 d | 1 |  |
|  | Repeated reports | 1 |  |
|  | Subtotal | 70 |  |
| All exclusion | **Total** | **2061** |  |
